# Pesticide Exposure and Depression among Male Private Pesticide Applicators in the Agricultural Health Study

**DOI:** 10.1289/ehp.1307450

**Published:** 2014-06-06

**Authors:** John D. Beard, David M. Umbach, Jane A. Hoppin, Marie Richards, Michael C.R. Alavanja, Aaron Blair, Dale P. Sandler, Freya Kamel

**Affiliations:** 1 Department of Epidemiology, Gillings School of Global Public Health, University of North Carolina at Chapel Hill, Chapel Hill, North Carolina, USA; 2 Epidemiology Branch, and; 3 Biostatistics Branch, National Institute of Environmental Health Sciences, National Institutes of Health, Department of Health and Human Services, Research Triangle Park, North Carolina, USA; 4 Westat, Inc., Durham, North Carolina, USA; 5 Occupational and Environmental Epidemiology Branch, Division of Cancer Epidemiology and Genetics, National Cancer Institute, National Institutes of Health, Department of Health and Human Services, Rockville, Maryland, USA

## Abstract

Background: Pesticide exposure may be positively associated with depression. Few previous studies have considered the episodic nature of depression or examined individual pesticides.

Objective: We evaluated associations between pesticide exposure and depression among male private pesticide applicators in the Agricultural Health Study.

Methods: We analyzed data for 10 pesticide classes and 50 specific pesticides used by 21,208 applicators enrolled in 1993–1997 who completed a follow-up telephone interview in 2005–2010. We divided applicators who reported a physician diagnosis of depression (*n* = 1,702; 8%) into those who reported a previous diagnosis of depression at enrollment but not follow-up (*n* = 474; 28%), at both enrollment and follow-up (*n* = 540; 32%), and at follow-up but not enrollment (*n* = 688; 40%) and used polytomous logistic regression to estimate odds ratios (ORs) and 95% CIs. We used inverse probability weighting to adjust for potential confounders and to account for the exclusion of 3,315 applicators with missing covariate data and 24,619 who did not complete the follow-up interview.

Results: After weighting for potential confounders, missing covariate data, and dropout, ever-use of two pesticide classes, fumigants and organochlorine insecticides, and seven individual pesticides—the fumigants aluminum phosphide and ethylene dibromide; the phenoxy herbicide (2,4,5-trichlorophenoxy)acetic acid (2,4,5-T); the organochlorine insecticide dieldrin; and the organophosphate insecticides diazinon, malathion, and parathion—were all positively associated with depression in each case group, with ORs between 1.1 and 1.9.

Conclusions: Our study supports a positive association between pesticide exposure and depression, including associations with several specific pesticides.

Citation: Beard JD, Umbach DM, Hoppin JA, Richards M, Alavanja MCR, Blair A, Sandler DP, Kamel F. 2014. Pesticide exposure and depression among male private pesticide applicators in the Agricultural Health Study. Environ Health Perspect 122:984–991; http://dx.doi.org/10.1289/ehp.1307450

## Introduction

Exposure to pesticides, particularly organophosphate insecticides (OPs), may be positively associated with depression (Bazylewicz-Walczak et al. 1999; Beseler and Stallones 2008; Beseler et al. 2006, 2008; Mackenzie Ross et al. 2010; Onwuameze et al. 2013; Rehner et al. 2000; Salvi et al. 2003; Weisskopf et al. 2013; Wesseling et al. 2010). However, only a few of these studies were longitudinal (Bazylewicz-Walczak et al. 1999; Beseler and Stallones 2008; Onwuameze et al. 2013; Salvi et al. 2003)—an important consideration because many people with depression will recover and some may relapse (Colman and Ataullahjan 2010). The largest longitudinal study previously conducted (651 Colorado farmers and their spouses) assessed depression annually for three years using the Center for Epidemiological Studies-Depression Scale (CES-D) and found that individuals who reported past pesticide poisoning at baseline were twice as likely to be depressed during follow-up as those who did not (Beseler and Stallones 2008). That study, however, did not evaluate associations with chronic exposure in the absence of poisoning or to specific pesticides.

The Agricultural Health Study (AHS) is a prospective cohort study, including 52,394 licensed private pesticide applicators (mostly farmers), designed to assess associations between agricultural exposures and health end points (Alavanja et al. 1996). We previously found a higher prevalence of depression among male applicators who reported past pesticide poisoning or use of pesticides from several different classes (Beseler et al. 2008). That study, however, used a cross-sectional design and did not examine specific pesticides. The aim of the current study is to assess associations between pesticide use and depression among male pesticide applicators in the AHS.

## Methods

*Study population and case definition*. From 1993 through 1997, pesticide applicators applying for or renewing their pesticide-use licenses at agricultural extension offices in Iowa and North Carolina were invited to enroll in the AHS (Alavanja et al. 1996). A total of 52,394 private applicators (84% of those eligible) enrolled by returning the enrollment questionnaire. An additional baseline questionnaire, the farmer questionnaire, was sent home with all enrolled applicators but returned by only 22,916 (44%). Applicators who returned the farmer questionnaire were older than those who did not, but generally similar otherwise (Tarone et al. 1997). A follow-up telephone interview in 2005–2010, an average of 12.1 years after enrollment, included questions on depression.

**Figure 1:**
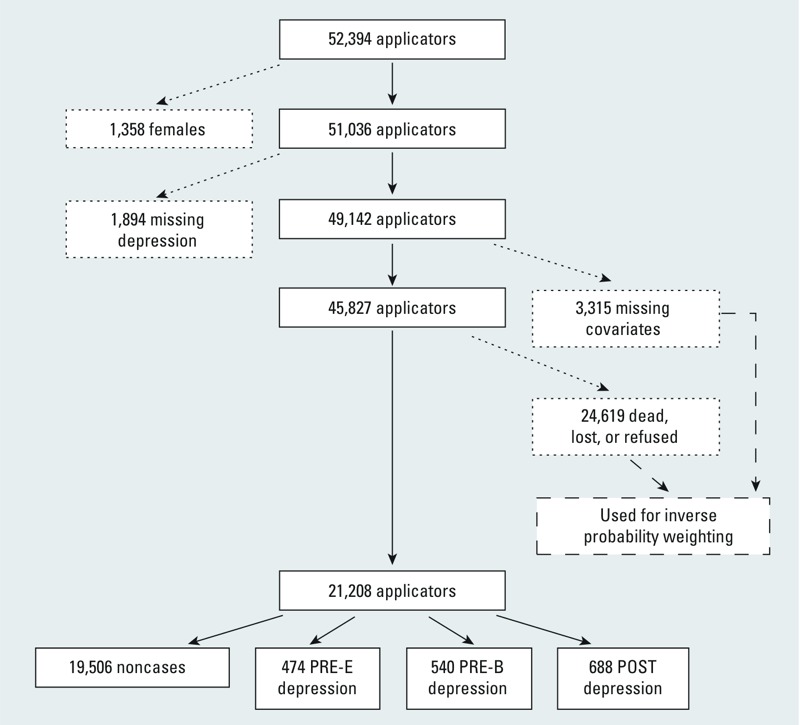
Flow diagram depicting the study population for an analysis of pesticide use and self-reported depression among male private pesticide applicators in the AHS. Solid boxes or lines represent individuals remaining in the study after each step; small-dashed boxes or lines represent individuals excluded after each step (see “Study population and case definition” for more details); large-dashed boxes or lines represent individuals incorporated into the analysis only indirectly via inverse probability weighting (see “Statistical analyses” for more details). Depression groups shown at the bottom of the diagram were defined as described in the text (see “Study population and case definition” for more details).

We excluded 6,567 applicators because they were female (1,358; 3%), were missing data on depression at enrollment and follow-up (1,894; 4%), or were missing covariate data (3,315; 6%); 45,827 (87%) applicators remained (Figure 1). In addition, 3,979 (8%) died before the follow-up interview and 20,640 (39%) did not complete it for other reasons. In total, we included 21,208 (40%) applicators in this analysis: 1,702 (8%) who reported ever receiving a physician’s diagnosis of depression (cases) and 19,506 (92%) who did not (noncases) (Figure 1).

Information on physician-diagnosed depression came from the enrollment and farmer questionnaires and the follow-up interview (AHS 2013). The enrollment questionnaire asked “Has a *doctor* ever told you that you had…[d]epression[?]” and the farmer questionnaire asked “Has a *DOCTOR* ever told you that you had (been diagnosed with)…[d]epression requiring medication or shock therapy?” We considered an applicator who responded affirmatively to either question to have a history of depression at enrollment. At follow-up, we asked “Have you ever been diagnosed with depression?” and “How old were you when you were first diagnosed with depression?” We considered any applicator who reported an age at diagnosis less than his age at enrollment to have a history of depression at enrollment regardless of his response to the enrollment depression questions.

We divided cases into three groups based on when the physician diagnosis of depression occurred (before or after enrollment) and on when it was reported via the AHS contacts (at enrollment, at follow-up, or both). The “pre-enrollment enrollment only” (PRE-E) group included 474 (28%) applicators who reported a previous diagnosis of depression at enrollment, but who did not confirm their pre-enrollment diagnosis at follow-up. The “pre-enrollment both” (PRE-B) group included 540 (32%) applicators who reported a previous diagnosis of depression at both enrollment and follow-up (*n* = 395), or who reported a previous diagnosis at follow-up only but with an age at diagnosis less than their age at enrollment (*n* = 145). The “post-enrollment” (POST) group included 688 (40%) applicators who reported a previous diagnosis of depression at follow-up but not at enrollment, and whose reported age at diagnosis equaled or exceeded their age at enrollment. Although both the PRE-E and PRE-B groups reported a diagnosis before enrollment, we treated them as separate outcomes in our analysis because we thought that the PRE-B group might be more likely to include men who had chronic depression, thus making them more likely to report a previous diagnosis at both time points, whereas the PRE-E group might not have reported a pre-enrollment diagnosis at follow-up because they did not experience depression during the follow-up period (12.1 years, on average). In addition, associations with pesticide use differed between the two groups. We cannot, however, confirm that the prevalence of depression over time differed between the two groups. It is also possible that PRE-E cases may have been less inclined to confirm their previous diagnosis of depression at follow-up because the interview was conducted via telephone, whereas depression information was collected at enrollment via self-administered paper questionnaires.

Some information on pesticide exposure was available only from the farmer questionnaire. Of the 21,208 applicators included in the analyses, 11,982 completed the farmer questionnaire. Of these, we classified 10,990 as noncases and 306 as PRE-E, 315 as PRE-B, and 371 as POST depression cases.

The AHS was approved by the institutional review boards (IRBs) of the National Institutes of Health and its contractors. The current analysis using coded data was exempted from review by the IRB of the University of North Carolina at Chapel Hill. All participants implied informed consent by returning the enrollment questionnaires and participating in the telephone interview.

*Exposure assessment*. At enrollment, applicators provided information on demographics, medical conditions, lifestyle, and pesticide use up until the time of enrollment by completing self-administered questionnaires (AHS 2013; Alavanja et al. 1996). We used three types of pesticide exposure variables: *a*) general exposure, *b*) use (personally mixed or applied) of pesticide classes, and *c*) use of individual pesticides. General exposure consisted of three variables: cumulative days of use of any pesticide, physician-diagnosed pesticide poisoning, and experiencing an incident of unusually high personal pesticide exposure (high pesticide exposure event). The latter two variables were available only for applicators who completed the farmer questionnaire. We calculated cumulative days of use of any pesticide as the product of reported duration (years) and frequency (days per year) and then categorized the result into four groups based on quartiles of use among all applicators. We created variables for ever-use of pesticides from four functional classes (fumigants, fungicides, herbicides, and insecticides) and six chemical classes (phenoxy and triazine herbicides, carbamates, and organochlorine, organophosphate, and pyrethroid insecticides) based on responses for individual pesticides. Use of 50 individual pesticides included ever-use and cumulative days of use. Information on ever-use was collected via the enrollment questionnaire for all 50 pesticides, whereas information on duration and frequency, used to calculate cumulative days of use, was collected via the enrollment questionnaire for 22 pesticides and via the farmer questionnaire for the other 28. We calculated cumulative days of use for individual pesticides as the product of duration and frequency variables and then categorized the result into four groups: nonusers plus users categorized at tertiles. For six pesticides, at least two of the 12 exposure-category by depression-group combinations had fewer than five cases, so we instead used three groups: nonusers plus users dichotomized at the median.

*Statistical analyses*. We had information from the enrollment questionnaire on potential confounders identified from previous literature: age, state, education, marital status, number of children in family, usual frequency of alcohol consumption per week in the past year, cigarette smoking, diabetes (an indication of chronic disease), farm size, and wearing chemical-resistant gloves when personally handling pesticides. For applicators who completed the farmer questionnaire, we also had information on number of doctor visits in the past year (an indication of general health), number of years lived or worked on a farm, working a job off a farm, and solvent (other than gasoline) exposure in the longest-held nonfarm job.

We used a directed acyclic graph (Greenland et al. 1999) to identify two minimally sufficient adjustment sets (MSASs) among potential confounders: *a*) age, alcohol consumption, diabetes, marital status, smoking, solvents, and state; and *b*) age, diabetes, education, and state (see Supplemental Material, Figure S1). This report used the second MSAS because it had less missing covariate information; the first MSAS gave similar results (data not shown).

For our main analyses, we used stabilized inverse probability weights to adjust for confounding and to account for the loss of 3,315 applicators with missing covariate data (in diabetes and education) and 24,619 applicators who did not complete the follow-up interview (Cole and Hernán 2008). For analyses involving information from the farmer questionnaire, we added a weight to account for the loss of 9,226 applicators who did not complete that questionnaire. We used polytomous logistic regression to estimate odds ratios (ORs) and 95% CIs for associations between pesticide exposure and depression within each case group, using noncases as the reference. These ORs apply to the population of 49,142 male applicators not missing data on depression at enrollment and at follow-up. We rounded all ORs and 95% CIs to the tenths place for presentation, and considered pesticide exposure to be “positively associated” with depression if the rounded lower 95% confidence limit for the OR was at least 1.0 or if the rounded OR was at least 1.3. We used Wald chi-square tests to test differences among case group–specific ORs at α = 0.1. We assessed linear trends for cumulative-days-of-use variables using the medians of each exposure category. We modeled the median category scores as continuous variables and scaled the trend ORs to interquartile range (IQR) increases in the original cumulative-days-of-use variables.

We used linear, logistic, or ordinal logistic regression, depending on the nature of the exposure variable, to calculate stabilized weights for confounding, missing covariate data, missing farmer questionnaire (if appropriate), and dropout for each exposure separately and then multiplied the three or four weights to obtain the overall stabilized weight (Cole and Hernán 2008; see also Supplemental Material, p. 4). In all models used to calculate the weights (see Supplemental Material, p. 4), we fit age as a restricted, quadratic spline with knots at 40, 48, and 57 years of age based on percentiles of the age distribution in all cases whereas diabetes, education, and state were modeled as shown in Table 1. We applied the overall stabilized weight to polytomous logistic regression models for depression that contained the exposure of interest as the only explanatory variable in the same way that sampling weights are applied when analyzing data from complex survey sampling designs (Cole and Hernán 2008). We calculated 95% CIs using robust variance estimates because using weights induces within-subject correlation (Hernán et al. 2000). We also conducted a sensitivity analysis without weighting; we used standard regression methods to adjust for potential confounding but without adjustment for potential biases from missing covariate data, missing farmer questionnaire, or dropout.

**Table 1:** Selected characteristics of male private pesticide applicators in the AHS.

Characteristic	Noncases [*n* (%)]	PRE-E^*a*^		PRE-B^*a*^		POST^*a*^		*p* for difference among ORs^*c*^
		Cases [*n* (%)]	Adjusted OR^*b*^ (95% CI)	Cases [*n* (%)]	Adjusted OR^*b*^ (95% CI)	Cases [*n* (%)]	Adjusted OR^*b*^ (95% CI)	
Total	19,506 (100)	474 (100)		540 (100)		688 (100)
Age at enrollment (years)
≤ 25	540 (3)	5 (1)	0.4 (0.2, 1.0)	7 (1)	0.5 (0.2, 1.0)	9 (1)	0.4 (0.2, 0.8)
26–35	2,879 (15)	25 (5)	0.4 (0.2, 0.6)	36 (7)	0.5 (0.3, 0.7)	119 (17)	1.0 (0.8, 1.3)
36–45	5,856 (30)	136 (29)	Reference	158 (29)	Reference	238 (35)	Reference
46–55	4,909 (25)	143 (30)	1.3 (1.0, 1.6)	177 (33)	1.3 (1.1, 1.7)	184 (27)	0.9 (0.7, 1.1)
56–65	3,902 (20)	120 (25)	1.3 (1.1, 1.7)	118 (22)	1.1 (0.9, 1.4)	96 (14)	0.6 (0.5, 0.8)
> 65	1,420 (7)	45 (9)	1.4 (1.0, 2.0)	44 (8)	1.2 (0.8, 1.7)	42 (6)	0.7 (0.5, 1.0)	< 0.01
State of residence
Iowa	13,520 (69)	329 (69)	Reference	384 (71)	Reference	460 (67)	Reference
North Carolina	5,986 (31)	145 (31)	0.9 (0.8, 1.1)	156 (29)	0.9 (0.7, 1.1)	228 (33)	1.2 (1.0, 1.4)	0.04
Education level
≤ Some high school or something else	1,343 (7)	48 (10)	1.4 (1.0, 1.9)	44 (8)	1.2 (0.8, 1.6)	45 (7)	1.1 (0.8, 1.5)
High school graduate or GED	9,045 (46)	213 (45)	Reference	251 (46)	Reference	314 (46)	Reference
1–3 years of vocational education beyond high school, some college, or college graduate	8,357 (43)	192 (41)	1.1 (0.9, 1.3)	226 (42)	1.0 (0.9, 1.2)	297 (43)	1.0 (0.8, 1.1)
≥ 1 years of graduate or professional school	761 (4)	21 (4)	1.1 (0.7, 1.7)	19 (4)	0.9 (0.5, 1.4)	32 (5)	1.2 (0.8, 1.7)	0.79
Ever diagnosed with diabetes
No	19,051 (98)	450 (95)	Reference	516 (96)	Reference	665 (97)	Reference
Yes	455 (2)	24 (5)	1.9 (1.2, 2.9)	24 (4)	1.8 (1.2, 2.7)	23 (3)	1.6 (1.0, 2.5)	0.84
Abbreviations: GED, General Equivalency Diploma; POST, post-enrollment; PRE-B, pre-enrollment both; PRE-E, pre-enrollment enrollment only. ^***a***^Cases were divided into three groups based on when the physician diagnosis of depression occurred (before or after enrollment) and on when it was reported via the AHS contacts (at enrollment, at follow-up, or both). The PRE-E group included applicators who reported a previous diagnosis of depression at enrollment, but who did not confirm their pre-enrollment diagnosis at follow-up. The PRE-B group included applicators who reported a previous diagnosis of depression at both enrollment and follow-up, or who reported a previous diagnosis at follow-up only but with an age at diagnosis less than their age at enrollment. The POST group included applicators who reported a previous diagnosis of depression at follow-up but not enrollment, and whose reported age at diagnosis equaled or exceeded their age at enrollment. ^***b***^Adjusted for age at enrollment (modeled with a cubic polynomial) and state of residence. ^***c***^Differences among case group–specific ORs tested via Wald chi-square tests.								

We used four criteria to evaluate the appropriateness of the weights used in our analyses: *a*) nearness of the mean weight to one, *b*) number of extreme weights (e.g., < 0.05 or > 20), *c*) positivity, and *d*) bias–variance (validity–precision) tradeoff (Cole and Hernán 2008). We did not consider the c-statistic, Hosmer–Lemeshow statistic, or any other measure of goodness-of-fit to select variables for inclusion in our models for the weights because doing so can lead to bias (from unbalanced confounders or balanced nonconfounders including instrumental variables), reduced precision, nonpositivity, and/or restricted inference (Westreich et al. 2011). To informally assess the bias–variance tradeoff (Winer 1978), we progressively truncated the overall stabilized weights by resetting weights less (or greater) than a certain percentile to the value of that percentile (Cole and Hernán 2008). Regarding the ORs derived from the untruncated weights as the “true” values, we informally evaluated bias–variance tradeoff by evaluating how features of both the weights and the corresponding ORs changed with increasing truncation. We considered nearness of the mean weight to one, reduction in number of extreme weights, and a balance between increased “bias” and reduced variance in the estimated ORs (Cole and Hernán 2008). Truncating the overall stabilized weights at the first and 99th percentiles appeared to be the best balance of validity and precision and mitigated problems identified by all of the criteria in this analysis.

We conducted several additional sensitivity analyses. We augmented models for ever-use of pesticide classes or individual pesticides by adding potentially confounding variables one at a time in models for all the different types of weights. These variables were number of children, doctor visits in the past year, farm size, use of chemical-resistant gloves, and cumulative lifetime days of use of any pesticide. We included all variables in Table 1 and in Supplemental Material, Table S1, in models for the dropout weights to evaluate whether there were selection effects beyond that captured by the covariates in the second MSAS. To account for correlations between use of different pesticides, we added the pesticide that was most strongly correlated with the pesticide of interest to models for the weights. We refit models excluding applicators who reported physician-diagnosed pesticide poisoning to evaluate whether or not results were driven by pesticide poisoning. Finally, we evaluated effect measure modification by state or by use of chemical-resistant gloves using the likelihood ratio test at α = 0.1. We performed all analyses via SAS version 9.2 (SAS Institute Inc., Cary, NC).

## Results

After adjustment for age at enrollment and state of residence, the odds of depression were higher in each case group for applicators who were past cigarette smokers compared with those who never smoked, who reported at least one visit to a medical doctor in the past year compared with no visits, and who reported a previous diagnosis of diabetes compared with none (Table 1; see also Supplemental Material, Table S1). For age, state, marital status, doctor visits in the past year, and solvent (other than gasoline) exposure in the longest-held nonfarm job, ORs for POST depression were generally different from ORs for PRE-E and PRE-B depression, whereas the latter two were generally similar (Table 1; see also Supplemental Material, Table S1).

The mean weight of all truncated overall stabilized weights was approximately one except that for the categorical version of cumulative days of carbaryl use (mean weight = 1.28). There were no extreme weights (see Supplemental Material, Tables S2–S4).

After weighting for age, diabetes diagnosis, education, state, missing covariate data, missing farmer questionnaire (where appropriate), and dropout, depression was positively associated with cumulative days of use of any pesticide, physician-diagnosed pesticide poisoning, and ever experiencing a high pesticide exposure event among PRE-E and PRE-B cases, but not among POST cases (Table 2). In each case group, depression was positively associated with ever-use of fumigants as a class and organochlorine insecticides as a class as well as the specific fumigants aluminum phosphide and ethylene dibromide; the phenoxy herbicide (2,4,5-trichlorophenoxy)acetic acid (2,4,5-T); the organochlorine insecticide dieldrin; and the OPs diazinon, malathion, and parathion (Table 3).

**Table 2:** Pesticide use and self-reported depression among male private pesticide applicators in the AHS.

Variable	Noncases [*n* (%)]	PRE-E^*a*^		PRE-B^*a*^		POST^*a*^		*p* for difference among ORs^*c*^
		Cases [*n* (%)]	IP-weighted OR^*b*^ (95% CI)	Cases [*n* (%)]	IP-weighted OR^*b*^ (95% CI)	Cases [*n* (%)]	IP-weighted OR^*b*^ (95% CI)	
Total	19,506 (100)	474 (100)		540 (100)		688 (100)
Cumulative days personally mixed or applied pesticides^*d*^
≤ 56 (median = 24.5)	4,520 (23)	79 (17)	Reference	102 (19)	Reference	164 (24)	Reference
57–225 (median = 116.0)	6,876 (35)	164 (35)	1.2 (0.9, 1.6)	189 (35)	1.1 (0.8, 1.4)	223 (32)	0.9 (0.7, 1.1)
226–457 (median = 369.8)	4,139 (21)	107 (23)	1.4 (1.0, 1.9)	129 (24)	1.3 (1.0, 1.8)	170 (25)	1.1 (0.9, 1.4)
> 457 (median = 767.3)	3,968 (20)	124 (26)	1.6 (1.2, 2.2)	120 (22)	1.3 (1.1, 1.7)	131 (19)	0.9 (0.7, 1.2)	0.10
Missing	3	0		0		0
Trend (IQR = 401.3)^*e*^			1.3 (1.1, 1.4)		1.1 (1.0, 1.3)		1.0 (0.9, 1.1)	0.03
Ever diagnosed with pesticide poisoning^*f*^
No	10,656 (98)	274 (90)	Reference	293 (95)	Reference	362 (98)	Reference
Yes	206 (2)	29 (10)	4.2 (2.7, 6.6)	16 (5)	2.5 (1.4, 4.4)	7 (2)	1.0 (0.4, 2.4)	0.01
Missing	128	3		6		2
Ever experienced an incident of unusually high personal pesticide exposure^*f*^
No	9,093 (85)	215 (72)	Reference	214 (71)	Reference	296 (83)	Reference
Yes	1,642 (15)	84 (28)	2.3 (1.8, 3.1)	86 (29)	2.2 (1.6, 2.9)	60 (17)	1.1 (0.8, 1.5)	< 0.01
Missing	255	7		15		15		
Abbreviations: IP, inverse probability; POST, post-enrollment; PRE-B, pre-enrollment both; PRE-E, pre-enrollment enrollment only. ^***a***^See Table 1 for a description of the three case groups. ^***b***^Weights were adjusted for age at enrollment (modeled with a restricted, quadratic spline with knots at 40, 48, and 57 years of age based on percentiles of the age distribution in cases), ever diagnosed with diabetes, education level, state of residence, not missing covariate data (conditional on age, state, the exposure, and pairwise interaction terms between each covariate and the exposure), and not dropping out of the AHS cohort (conditional on age, diabetes, education, state, the exposure, and pairwise interaction terms between each covariate and the exposure). 95% CIs were calculated with robust variance estimates. ^***c***^Differences among case group–specific ORs were tested via Wald chi-square tests. ^***d***^Category boundaries were set at quartiles of cumulative days of pesticide use among all male private pesticide applicators. ^***e***^We used within-category medians and scaled the OR to an IQR-unit (days) increase in cumulative days of pesticide use among all male private pesticide applicators. ^***f***^Data were available only for 11,982 applicators who completed the farmer questionnaire. Weights were additionally adjusted for completing the farmer questionnaire (conditional on age, diabetes, education, and state).								

**Table 3:** Ever-use of pesticide classes and specific pesticides and self-reported depression among male private pesticide applicators in the AHS.

Ever personally mixed or applied	Noncases^*a*^ [*n* (%)]	PRE-E^*b*^		PRE-B^*b*^		POST^*b*^		*p* for difference among ORs^*e*^
		Cases^*a*^ [*n* (%)]	IP-weighted OR^*c,d*^ (95% CI)	Cases^*a*^ [*n* (%)]	IP-weighted OR^*c,d*^ (95% CI)	Cases^*a*^ [*n* (%)]	IP-weighted OR^*c,d*^ (95% CI)	
Total	19,506 (100)	474 (100)		540 (100)		688 (100)
Fumigants	4,363 (23)	131 (29)	1.4 (1.1, 1.8)	166 (32)	1.8 (1.5, 2.3)	177 (27)	1.2 (1.0, 1.5)	0.03
Aluminum phosphide	940 (5)	32 (7)	1.4 (0.9, 2.0)	38 (7)	1.3 (0.9, 1.9)	49 (8)	1.6 (1.1, 2.2)	0.75
Carbon tetrachloride/carbon disulfide (80/20 mix)	1,164 (6)	46 (10)	1.8 (1.3, 2.6)	53 (11)	1.9 (1.4, 2.7)	44 (7)	1.2 (0.8, 1.7)	0.11
Ethylene dibromide	676 (4)	24 (5)	1.7 (1.0, 2.7)	25 (5)	1.5 (1.0, 2.4)	29 (5)	1.3 (0.9, 2.1)	0.79
Methyl bromide	2,853 (15)	75 (16)	1.2 (0.7, 1.9)	90 (17)	1.6 (1.0, 2.4)	109 (16)	1.2 (0.8, 1.8)	0.57
Fungicides	6,850 (36)	184 (40)	1.2 (1.0, 1.5)	213 (41)	1.3 (1.1, 1.6)	256 (39)	1.1 (0.9, 1.3)	0.33
Benomyl^*f*^	1,793 (10)	50 (11)	1.5 (1.0, 2.2)	48 (9)	1.1 (0.7, 1.7)	70 (11)	1.3 (0.9, 1.8)	0.67
Captan	2,301 (12)	62 (14)	1.2 (0.9, 1.5)	86 (17)	1.4 (1.1, 1.8)	90 (14)	1.2 (0.9, 1.5)	0.52
Chlorothalonil	1,326 (7)	31 (7)	0.9 (0.5, 1.5)	43 (8)	1.3 (0.8, 2.0)	55 (8)	1.2 (0.8, 1.7)	0.58
Maneb/mancozeb	1,775 (10)	50 (11)	1.3 (0.8, 2.0)	51 (10)	1.2 (0.7, 1.8)	65 (10)	1.2 (0.8, 1.8)	0.95
Metalaxyl	4,157 (22)	120 (27)	1.5 (1.1, 1.9)	122 (24)	1.3 (1.0, 1.7)	151 (23)	1.0 (0.8, 1.3)	0.12
Ziram	276 (2)	10 (2)	1.6 (0.8, 3.1)	5 (1)	0.8 (0.3, 2.0)	12 (2)	1.4 (0.8, 2.6)	0.46
Herbicides	19,086 (98)	469 (99)	1.6 (0.7, 4.0)	533 (99)	1.8 (0.8, 3.9)	677 (99)	1.1 (0.6, 2.1)	0.62
Alachlor	10,526 (56)	287 (63)	1.3 (1.0, 1.6)	325 (62)	1.2 (1.0, 1.4)	384 (59)	1.1 (0.9, 1.3)	0.61
Butylate	6,338 (34)	162 (36)	1.1 (0.9, 1.3)	196 (39)	1.1 (0.9, 1.3)	234 (36)	1.1 (0.9, 1.3)	0.80
Chlorimuron-ethyl	7,077 (38)	160 (36)	0.9 (0.8, 1.2)	199 (39)	1.1 (0.9, 1.3)	261 (40)	1.0 (0.9, 1.2)	0.59
Dicamba	10,237 (55)	248 (54)	0.9 (0.7, 1.1)	292 (57)	1.0 (0.8, 1.2)	365 (57)	1.0 (0.8, 1.2)	0.74
EPTC	4,013 (22)	113 (25)	1.2 (0.9, 1.5)	105 (21)	0.9 (0.7, 1.2)	156 (24)	1.0 (0.8, 1.3)	0.44
Glyphosate	15,053 (78)	376 (80)	1.2 (0.9, 1.6)	426 (79)	1.1 (0.9, 1.4)	540 (79)	1.1 (0.9, 1.3)	0.80
Imazethapyr	8,480 (46)	207 (46)	1.0 (0.8, 1.3)	220 (43)	0.9 (0.7, 1.1)	304 (47)	1.1 (0.9, 1.3)	0.42
Metolachlor	9,121 (49)	229 (51)	1.1 (0.9, 1.3)	231 (45)	0.8 (0.7, 1.0)	311 (48)	1.0 (0.8, 1.1)	0.20
Paraquat	4,402 (24)	120 (26)	1.2 (1.0, 1.5)	123 (25)	1.1 (0.9, 1.4)	158 (24)	1.1 (0.9, 1.3)	0.77
Pendimethalin	8,372 (45)	218 (48)	1.2 (1.0, 1.4)	217 (42)	0.9 (0.8, 1.1)	282 (43)	0.9 (0.8, 1.1)	0.09
Petroleum oil	9,408 (51)	260 (58)	1.3 (1.1, 1.6)	285 (57)	1.2 (0.9, 1.5)	336 (52)	1.0 (0.9, 1.2)	0.11
Trifluralin	10,286 (55)	266 (59)	1.2 (1.0, 1.5)	299 (58)	1.1 (0.9, 1.3)	363 (56)	1.1 (0.9, 1.3)	0.63
Phenoxy herbicides	15,742 (82)	391 (84)	1.1 (0.9, 1.5)	456 (86)	1.3 (1.0, 1.7)	541 (80)	0.9 (0.8, 1.1)	0.11
2,4-D	15,371 (79)	378 (81)	1.1 (0.8, 1.4)	442 (82)	1.2 (0.9, 1.5)	526 (78)	1.0 (0.8, 1.2)	0.45
2,4,5-T	4,517 (24)	157 (35)	1.6 (1.3, 2.0)	178 (35)	1.6 (1.3, 1.9)	157 (24)	1.2 (1.0, 1.5)	0.10
2,4,5-TP	1,841 (10)	71 (16)	1.7 (1.3, 2.2)	73 (14)	1.7 (1.3, 2.2)	67 (11)	1.1 (0.9, 1.5)	0.07
Triazine herbicides	15,768 (82)	393 (84)	1.1 (0.8, 1.5)	445 (83)	1.0 (0.8, 1.3)	556 (82)	1.1 (0.8, 1.3)	0.91
Atrazine	14,554 (75)	372 (79)	1.2 (1.0, 1.6)	415 (77)	1.0 (0.8, 1.3)	511 (75)	1.0 (0.8, 1.2)	0.44
Cyanazine	8,399 (45)	233 (51)	1.3 (1.0, 1.6)	258 (50)	1.1 (0.9, 1.3)	304 (46)	1.1 (0.9, 1.4)	0.55
Metribuzin	9,061 (49)	236 (52)	1.1 (0.9, 1.4)	264 (52)	1.0 (0.9, 1.3)	322 (49)	1.0 (0.9, 1.2)	0.83
Insecticides	18,379 (95)	458 (97)	1.3 (0.7, 2.2)	510 (95)	1.0 (0.6, 1.5)	655 (97)	1.5 (1.0, 2.4)	0.34
Carbamates^*f*^	13,037 (68)	335 (71)	1.0 (0.8, 1.3)	389 (73)	1.0 (0.8, 1.3)	475 (70)	1.1 (0.9, 1.3)	0.95
Aldicarb	1,891 (10)	42 (9)	0.9 (0.6, 1.5)	52 (10)	1.4 (1.0, 2.2)	81 (13)	1.4 (1.0, 1.9)	0.28
Carbaryl	10,984 (58)	295 (64)	1.2 (0.9, 1.5)	336 (64)	1.2 (1.0, 1.5)	411 (62)	1.1 (0.9, 1.4)	0.87
Carbofuran	5,576 (30)	153 (34)	1.2 (1.0, 1.5)	181 (35)	1.2 (1.0, 1.5)	180 (28)	0.9 (0.8, 1.1)	0.14
Organochlorine insecticides	10,316 (55)	333 (72)	1.9 (1.5, 2.4)	334 (64)	1.2 (1.0, 1.4)	368 (56)	1.2 (1.0, 1.5)	0.01
Aldrin	3,991 (22)	140 (31)	1.4 (1.1, 1.9)	159 (31)	1.5 (1.2, 1.9)	137 (21)	1.2 (0.9, 1.5)	0.36
Chlordane	5,321 (28)	185 (41)	1.6 (1.3, 2.0)	179 (35)	1.3 (1.0, 1.6)	185 (29)	1.1 (0.9, 1.3)	0.03
DDT	5,152 (28)	174 (38)	1.8 (1.4, 2.3)	175 (34)	1.3 (1.0, 1.7)	143 (22)	1.0 (0.7, 1.3)	0.01
Dieldrin	1,476 (8)	56 (13)	1.6 (1.1, 2.3)	59 (12)	1.6 (1.1, 2.2)	48 (7)	1.3 (0.9, 1.8)	0.63
Heptachlor	3,354 (18)	131 (29)	1.6 (1.3, 2.1)	126 (25)	1.3 (1.0, 1.7)	100 (16)	1.0 (0.8, 1.3)	0.04
Lindane	4,053 (22)	146 (32)	1.6 (1.3, 2.0)	141 (28)	1.3 (1.0, 1.6)	152 (23)	1.2 (0.9, 1.4)	0.08
Toxaphene	2,899 (16)	97 (22)	1.5 (1.1, 1.9)	110 (22)	1.5 (1.2, 1.9)	104 (16)	1.1 (0.9, 1.4)	0.12
Organophosphate insecticides	17,563 (91)	442 (94)	1.6 (1.1, 2.3)	494 (92)	1.2 (0.8, 1.7)	629 (93)	1.3 (1.0, 1.8)	0.56
Chlorpyrifos	8,457 (44)	221 (47)	1.2 (1.0, 1.4)	272 (50)	1.3 (1.1, 1.5)	300 (44)	1.0 (0.9, 1.2)	0.10
Coumaphos	1,799 (10)	57 (13)	1.2 (0.9, 1.7)	63 (13)	1.3 (1.0, 1.7)	54 (9)	0.8 (0.6, 1.1)	0.03
Diazinon	6,211 (33)	182 (40)	1.4 (1.1, 1.7)	207 (41)	1.3 (1.1, 1.6)	235 (36)	1.2 (1.0, 1.4)	0.51
Dichlorvos	1,856 (12)	61 (14)	1.1 (0.8, 1.5)	96 (19)	1.6 (1.3, 2.1)	99 (15)	1.3 (1.0, 1.6)	0.11
Fonofos	4,396 (24)	132 (29)	1.3 (1.0, 1.7)	144 (28)	1.1 (0.9, 1.4)	146 (23)	0.9 (0.7, 1.2)	0.18
Malathion	13,941 (74)	369 (80)	1.3 (1.0, 1.7)	410 (79)	1.2 (1.0, 1.6)	503 (76)	1.1 (1.0, 1.4)	0.62
Parathion	2,903 (16)	102 (23)	1.5 (1.2, 1.9)	95 (19)	1.2 (1.0, 1.6)	116 (18)	1.3 (1.0, 1.6)	0.51
Phorate	6,523 (35)	191 (42)	1.3 (1.0, 1.6)	196 (38)	1.0 (0.8, 1.2)	228 (35)	1.0 (0.8, 1.2)	0.25
Terbufos	7,746 (42)	223 (50)	1.4 (1.1, 1.7)	240 (47)	1.2 (1.0, 1.4)	265 (41)	1.0 (0.8, 1.2)	0.07
Trichlorfon	123 (1)	5 (1)	1.5 (0.6, 3.7)	2 (1)	*—*^*g*^	1 (< 1)	*—*^*g*^	*—*^*g*^
Pyrethroid insecticides	4,805 (26)	128 (28)	1.2 (1.0, 1.5)	146 (28)	1.1 (0.9, 1.4)	164 (25)	0.9 (0.8, 1.1)	0.17
Permethrin (for animals)	2,841 (15)	78 (17)	1.2 (0.9, 1.5)	87 (17)	1.0 (0.8, 1.4)	104 (16)	1.0 (0.8, 1.3)	0.74
Permethrin (for crops)	2,539 (14)	68 (15)	1.2 (0.9, 1.6)	85 (17)	1.3 (1.0, 1.7)	82 (13)	0.9 (0.7, 1.2)	0.09
Abbreviations: 2,4-D, (2,4-dichlorophenoxy)acetic acid; 2,4,5-T, (2,4,5-trichlorophenoxy)acetic acid; 2,4,5-TP, (*RS*)-2-(2,4,5-trichlorophenoxy)propionic acid; DDT, 1,1,1-trichloro-2,2-bis(4-chlorophenyl)ethane; EPTC, *S*-ethyl dipropyl(thiocarbamate); IP, inverse probability; POST, post-enrollment; PRE-B, pre-enrollment both; PRE-E, pre-enrollment enrollment only. ^***a***^Information for specific pesticides was missing for < 1–6% of male private pesticide applicators. ^***b***^See Table 1 for a description of the three case groups. ^***c***^Male private pesticide applicators who did not use each pesticide class or specific pesticide were the reference. ^***d***^Weights were adjusted for age at enrollment (modeled with a restricted, quadratic spline with knots at 40, 48, and 57 years of age based on percentiles of the age distribution in cases), ever diagnosed with diabetes, education level, state of residence, not missing covariate data (conditional on age, state, the exposure, and pairwise interaction terms between each covariate and the exposure), and not dropping out of the AHS cohort (conditional on age, diabetes, education, state, the exposure, and pairwise interaction terms between each covariate and the exposure). 95% CIs were calculated with robust variance estimates. ^***e***^Differences among case group–specific ORs were tested via Wald chi-square tests. ^***f***^Benomyl is also included in carbamates. ^***g***^OR (95% CI) and *p* for difference not shown because fewer than five PRE-B or POST cases ever personally mixed or applied trichlorfon.								

Many pesticides were positively associated with depression in one or two, but not all three, case groups, but the ORs did not differ significantly (Table 3). Wald chi-square tests indicated that associations for ever-use of two pesticide classes and nine specific pesticides differed significantly at α = 0.1 among case groups. ORs for PRE-B depression were higher than those for PRE-E and POST depression for fumigants as a class, whereas ORs for PRE-E depression were higher than those for PRE-B and POST depression for organochlorine insecticides as a class (Table 3). For the nine specific pesticides, the most consistent finding was that ORs were elevated (lower 95% confidence limit ≥ 1.0 or OR ≥ 1.3) for PRE-E and PRE-B depression, but not for POST depression; this pattern was observed for the phenoxy herbicide (*RS*)-2-(2,4,5-trichlorophenoxy)propionic acid (2,4,5-TP); the organochlorine insecticides chlordane, 1,1,1-trichloro-2,2-bis(4-chlorophenyl)ethane (DDT), heptachlor, and lindane; and the OP terbufos (Table 3).

We observed positive trend ORs, based on the medians of each exposure category and scaled to IQR increases in the original cumulative-days-of-use variables, for associations between depression and cumulative days of use of the fumigants ethylene dibromide and methyl bromide; the fungicide captan; and the organochlorine insecticide lindane in each case group (see Supplemental Material, Table S5). For none of these agents, however, were the categorical ORs monotonically increasing in each case group (see Supplemental Material, Table S5). We also observed positive trend ORs for several other pesticides in at least one case group and several pesticides had significantly different trend ORs at α = 0.1 among case groups (see Supplemental Material, Table S5).

Augmenting models for ever-use of pesticide classes or individual pesticides by including additional variables (number of children, doctor visits in the past year, farm size, use of chemical-resistant gloves, cumulative lifetime days of use of any pesticide, or the pesticide that was most strongly correlated with the pesticide of interest) one at a time in models for all the different types of weights did not meaningfully change results, nor did including all variables in Table 1 and Supplemental Material, Table S1, in the models for the dropout weights (data not shown). Excluding applicators who reported physician-diagnosed pesticide poisoning did not change results (data not shown). We saw no consistent evidence of effect measure modification by state or by use of chemical-resistant gloves (data not shown). Finally, results were similar when we used standard regression methods (see Supplemental Material, Tables S6–S7).

## Discussion

We found positive associations between use of some pesticides and depression among male private pesticide applicators in the AHS. Depression was positively associated in each case group with ever-use of two pesticide classes, fumigants and organochlorine insecticides, as well as with ever-use of seven individual pesticides: the fumigants aluminum phosphide and ethylene dibromide; the phenoxy herbicide 2,4,5-T; the organochlorine insecticide dieldrin; and the OPs diazinon, malathion, and parathion. Positive relationships between depression and cumulative days of use were evident, though nonmonotonic, in each case group for the fumigants ethylene dibromide and methyl bromide, the fungicide captan, and the organochlorine insecticide lindane.

Positive associations between depression and acute, high-intensity pesticide exposures, such as pesticide poisoning or high pesticide exposure events, were reported previously in a longitudinal study of 651 Colorado farmers and their spouses (Beseler and Stallones 2008) and cross-sectional studies of 208 Costa Rican banana plantation workers (Wesseling et al. 2010), and 17,585 male private pesticide applicators (Beseler et al. 2008) and 29,074 wives in the AHS (Beseler et al. 2006). In our study, depression was positively associated with physician-diagnosed pesticide poisoning and high pesticide exposure events among PRE-E and PRE-B cases, but not among POST cases.

Previous studies have observed positive associations between depression and exposure to any pesticides or to some pesticide classes, particularly OPs: a follow-up study in Brazil that compared 25 agricultural workers assessed after 3 months of OP exposure with themselves assessed again after 3 months of no OP exposure (Salvi et al. 2003); a 3-month follow-up study in Poland that compared 26 OP-exposed greenhouse workers with 25 unexposed canteen, kitchen, and administrative workers (Bazylewicz-Walczak et al. 1999); a 3-year follow-up study of 257 farm operators in Iowa that compared those exposed to pesticides with those who were not (Onwuameze et al. 2013); a cross-sectional study in England that compared 127 current and retired sheep dippers exposed to OPs with 78 unexposed current and retired police officers (Mackenzie Ross et al. 2010); and a cross-sectional study of 17,585 male private pesticide applicators in the AHS that separately compared those exposed to any pesticide or to seven pesticide classes (carbamates, fumigants, fungicides, herbicides, insecticides, organochlorine insecticides, OPs) with those who were not (Beseler et al. 2008). A study of 567 agricultural workers in France that evaluated exposure to any pesticide, three pesticide classes, or 13 herbicide families, using no exposure to the pesticide class/family in question as the reference, reported positive associations between depression and exposure to herbicides in general and dinitrophenol herbicides, but not exposure to any pesticide, fungicides, insecticides, or the other 12 herbicide families (Weisskopf et al. 2013). In contrast, a cross-sectional survey of 9,844 sheep dippers in England and Wales that used no exposure to any pesticides as the common reference found no association between depression and use of sheep dip (usually diazinon or other OPs), other insecticides, herbicides, fungicides, or wood preservatives (Solomon et al. 2007). In our study, depression was positively associated with cumulative days of use of any pesticide among PRE-E and PRE-B cases, ever-use of the pesticides classes fumigants and organochlorine insecticides in each case group, and ever-use of several other pesticide classes, including OPs, in at least one case group. Results appeared to be independent of pesticide poisoning, because we observed similar results when we excluded applicators who reported physician-diagnosed pesticide poisoning (data not shown).

Only one previous study evaluated the association between depression and a specific pesticide, finding a cross-sectional association between parathion exposure and CES-D scores indicative of clinical depression among 115 adults in Jackson County, Mississippi (Rehner et al. 2000). We found that ever-use or trend versions of cumulative lifetime days of use of several individual pesticides, including parathion, were positively associated with depression.

In general, we observed fewer positive associations between pesticide use and depression among POST cases than among PRE-E or PRE-B cases. Reverse causation—where depression increases exposure, perhaps through careless handling of pesticides—is unlikely to explain the differences in associations among case groups because use of chemical-resistant gloves was not inversely associated with depression after adjustment for age and state, and because including use of chemical-resistant gloves in models for the weights did not change results. Alternatively, differences among case group–specific associations might be attributable to exposure being evaluated closer to first reported diagnosis of depression for PRE-E and PRE-B cases than for POST cases, which could be particularly important for pesticides, such as organochlorine insecticides, with marked secular trends in use. Using information on past instead of ongoing pesticide use could have obscured associations with POST depression. Differences among case group–specific associations might be attributable to residual confounding from observed differences in personal characteristics or in cumulative days of use of any pesticide among case groups; for example, the average cumulative days of use of any pesticide reported by POST cases was 343 compared with 424 for PRE-E and 387 for PRE-B cases (Kruskal–Wallis *p* = 0.02). Finally, although we asked about ever-diagnosis of depression at both enrollment and follow-up, some PRE-E depression cases were likely misclassified because they did not report a previous diagnosis at follow-up; in other words, they should have been classified as PRE-B cases. Possible reasons for this omission include recovering from depression before the follow-up interview (which was administered 12.1 years, on average, after enrollment) or, due to the sensitive nature of mental health conditions, being less inclined to confirm a previous diagnosis of depression because the follow-up interview was conducted via telephone, whereas depression information was collected at enrollment via self-administered paper questionnaires. We cannot, however, confirm either of these possibilities. Despite this possible misclassification, we analyzed PRE-E depression as a separate case group because the number of applicators in this group was large (*n* = 474) and associations with pesticide use differed from those observed with PRE-B depression.

We used three strategies to account for exposure to multiple pesticides. First, we grouped individual pesticides into 10 pesticide classes (4 functional, 6 chemical) because the pesticide that was most strongly correlated with the pesticide of interest was often in the same class. We also conducted sensitivity analyses in which we additionally weighted for cumulative days of use of any pesticide or for the pesticide that was most strongly correlated with the pesticide of interest. Although neither strategy meaningfully changed our results (data not shown), we cannot rule out the possibility that associations between depression and use of individual pesticides were confounded by use of other pesticides.

We used inverse probability weighting to adjust for potential confounding and for potential biases from missing covariate data, missing farmer questionnaires, or dropout. One limitation of inverse probability weighting is that residual confounding, missing data bias, and/or selection bias could still occur. In addition, c-statistics for the dropout models, while not used to select variables for inclusion in our models for the weights, ranged from 0.60 to 0.61, which suggests that dropout in the AHS is mostly random or that our models did not predict dropout well. The former seems more likely because Montgomery et al. (2010) found that applicators who reported physician-diagnosed depression at enrollment were equally likely to drop out of the AHS before the first follow-up interview in 1998–2003 as applicators who did not report depression (OR = 0.92; 95% CI: 0.82, 1.02 after adjustment for age, state, education, and smoking).

Our information on pesticide use was self-reported and could be misclassified. Using data from orchardists in Washington State reported during the year of use as the gold standard, Engel et al. (2001) found sensitivities for reporting ever-use of pesticides 25 years later were 1.00 for any pesticides, 0.87–1.00 for pesticides classes included in our study, and 0.80–0.94 for individual pesticides included in our study. A case–control study of cancer in Montreal, Canada, found the specificity of self-reported ever-exposure to pesticides or fertilizers was 0.95 when compared with expert assessment (Fritschi et al. 1996). In a reliability study of a subset of AHS applicators in Iowa who completed the enrollment questionnaire twice 1 year apart, percent exact agreement for ever-use of 10 individual pesticides ranged from 0.79 to 0.88 (Blair et al. 2002). Another study found that < 1–5% of AHS applicators overestimated duration of use of 19 individual pesticides relative to the years the pesticide active ingredients were first registered for use with the U.S. Environmental Protection Agency (Hoppin et al. 2002). The effect of depression on recall of past pesticide use is unknown. Cancer cases and controls, however, were found to report pesticide use with similar accuracy in a validation study in Kansas (Blair and Zahm 1993), and there is little evidence for differential recall in the self-reporting of occupational exposures among cases and controls of other diseases (Teschke et al. 2002).

We also relied on self-reports of ever physician-diagnosed depression. Using information from a validation study conducted in a cohort of university graduates in Spain, the calculated sensitivity and specificity of self-reported ever physician-diagnosed depression was 0.85 and 0.68, respectively, when the Structured Clinical Interview for the *Diagnostic and Statistical Manual of Mental Disorders, Fourth Edition*, was used as the gold standard (Sanchez-Villegas et al. 2008). In addition, associations we observed with pesticide poisoning and patient characteristics were similar to those reported in other studies, increasing confidence in the accuracy of our outcome. For example, depression was more common among applicators who were past smokers (Strine et al. 2008) or who had visited a medical doctor in the past year or had poorer health (Beseler and Stallones 2008). Therefore, the validity of self-reported ever physician-diagnosed depression in our study is likely good.

Our cohort is imperfect for longitudinal analyses of pesticide exposure and depression because we collected information on depression at only two points in time on average 12.1 years apart, and we assessed ever physician-diagnosed depression rather than current depression. Thus, we were unable to use longitudinal or life-course statistical methods.

Our study has several strengths, including its large size. Its prospective nature provided the opportunity to identify POST cases of depression as well as PRE-E and PRE-B cases. We had detailed information on applicators’ exposures, including general pesticide exposure, use of pesticide classes, and use of individual pesticides. We could control for many potential confounders and demonstrated the robustness of our results to additional potential confounders not included in the main models (data not shown). Finally, we used inverse probability weighting to adjust for potential biases from missing covariate data, missing farmer questionnaires, or dropout. Overall, the effect of missing data and dropouts on our results appeared to be small because results were similar when we used standard regression methods (see Supplemental Material, Tables S6–S7).

## Conclusions

Our study supports a positive association between depression and occupational pesticide use among applicators. Furthermore, it suggests several specific pesticides that deserve further investigation in animal studies and other human populations.

## Supplemental Material

(1.3 MB) PDF

## References

[r1] AHS (Agricultural Health Study). (2013). Questionnaires & Study Data.. http://aghealth.nih.gov/collaboration/questionnaires.html.

[r2] Alavanja MCR, Sandler D, McMaster S, Zahm S, McDonnell C, Lynch C (1996). The Agricultural Health Study.. Environ Health Perspect.

[r3] Bazylewicz-Walczak B, Majczakowa W, Szymczak M (1999). Behavioral effects of occupational exposure to organophosphorous pesticides in female greenhouse planting workers.. Neurotoxicology.

[r4] Beseler CL, Stallones L (2008). A cohort study of pesticide poisoning and depression in Colorado farm residents.. Ann Epidemiol.

[r5] Beseler C, Stallones L, Hoppin JA, Alavanja MC, Blair A, Keefe T (2006). Depression and pesticide exposures in female spouses of licensed pesticide applicators in the Agricultural Health Study cohort.. J Occup Environ Med.

[r6] Beseler CL Stallones L Hoppin JA Alavanja MC Blair A Keefe T 2008 Depression and pesticide exposures among private pesticide applicators enrolled in the Agricultural Health Study. Environ Health Perspect 116 1713 1719; 10.1289/ehp.11091 19079725 PMC2599768

[r7] Blair A, Tarone R, Sandler D, Lynch CF, Rowland A, Wintersteen W (2002). Reliability of reporting on life-style and agricultural factors by a sample of participants in the Agricultural Health Study from Iowa.. Epidemiology.

[r8] Blair A, Zahm SH (1993). Patterns of pesticide use among farmers: implications for epidemiologic research.. Epidemiology.

[r9] Cole SR, Hernán MA (2008). Constructing inverse probability weights for marginal structural models.. Am J Epidemiol.

[r10] Colman I, Ataullahjan A (2010). Life course perspectives on the epidemiology of depression.. Can J Psychiatry.

[r11] Engel LS, Seixas NS, Keifer MC, Longstreth WT, Checkoway H (2001). Validity study of self-reported pesticide exposure among orchardists.. J Expo Anal Environ Epidemiol.

[r12] Fritschi L, Siemiatycki J, Richardson L (1996). Self-assessed versus expert-assessed occupational exposures.. Am J Epidemiol.

[r13] Greenland S, Pearl J, Robins JM (1999). Causal diagrams for epidemiologic research.. Epidemiology.

[r14] Hernán MA, Brumback B, Robins JM (2000). Marginal structural models to estimate the causal effect of zidovudine on the survival of HIV-positive men.. Epidemiology.

[r15] Hoppin JA, Yucel F, Dosemeci M, Sandler DP (2002). Accuracy of self-reported pesticide use duration information from licensed pesticide applicators in the Agricultural Health Study.. J Expo Anal Environ Epidemiol.

[r16] Mackenzie Ross SJ, Brewin CR, Curran HV, Furlong CE, Abraham-Smith KM, Harrison V (2010). Neuropsychological and psychiatric functioning in sheep farmers exposed to low levels of organophosphate pesticides.. Neurotoxicol Teratol.

[r17] Montgomery MP, Kamel F, Hoppin JA, Beane Freeman LE, Alavanja MC, Sandler DP (2010). Effects of self-reported health conditions and pesticide exposures on probability of follow-up in a prospective cohort study.. Am J Ind Med.

[r18] Onwuameze OE, Paradiso S, Peek-Asa C, Donham KJ, Rautiainen RH (2013). Modifiable risk factors for depressed mood among farmers.. Ann Clin Psychiatry.

[r19] Rehner TA, Kolbo JR, Trump R, Smith C, Reid D (2000). Depression among victims of south Mississippi’s methyl parathion disaster.. Health Soc Work.

[r20] Salvi RM, Lara DR, Ghisolfi ES, Portela LV, Dias RD, Souza DO (2003). Neuropsychiatric evaluation in subjects chronically exposed to organophosphate pesticides.. Toxicol Sci.

[r21] Sanchez-Villegas A Schlatter J Ortuno F Lahortiga F Pla J Benito S 2008 Validity of a self-reported diagnosis of depression among participants in a cohort study using the Structured Clinical Interview for DSM-IV (SCID-I). BMC Psychiatry 8 43; 10.1186/1471-244X-8-43 18558014 PMC2447836

[r22] Solomon C, Poole J, Palmer KT, Peveler R, Coggon D (2007). Neuropsychiatric symptoms in past users of sheep dip and other pesticides.. Occup Environ Med.

[r23] Strine TW, Mokdad AH, Balluz LS, Gonzalez O, Crider R, Berry JT (2008). Depression and anxiety in the United States: findings from the 2006 Behavioral Risk Factor Surveillance System.. Psychiatr Serv.

[r24] Tarone RE, Alavanja MC, Zahm SH, Lubin JH, Sandler DP, McMaster SB (1997). The Agricultural Health Study: factors affecting completion and return of self-administered questionnaires in a large prospective cohort study of pesticide applicators.. Am J Ind Med.

[r25] Teschke K, Olshan AF, Daniels JL, De Roos AJ, Parks CG, Schulz M (2002). Occupational exposure assessment in case-control studies: opportunities for improvement.. Occup Environ Med.

[r26] Weisskopf MG, Moisan F, Tzourio C, Rathouz PJ, Elbaz A (2013). Pesticide exposure and depression among agricultural workers in France.. Am J Epidemiol.

[r27] Wesseling C, van Wendel de Joode B, Keifer M, London L, Mergler D, Stallones L (2010). Symptoms of psychological distress and suicidal ideation among banana workers with a history of poisoning by organophosphate or *n*-methyl carbamate pesticides.. Occup Environ Med.

[r28] Westreich D, Cole SR, Funk MJ, Brookhart MA, Stürmer T (2011). The role of the c-statistic in variable selection for propensity score models.. Pharmacoepidemiol Drug Saf.

[r29] Winer BJ (1978). Statistics and data analysis: trading bias for reduced mean squared error.. Annu Rev Psychol.

