# Supplementary material for: Pesticide Exposure and Depression among Male Private Pesticide Applicators in the Agricultural Health Study
Source: Environ Health Perspect. 2014 Jun 6;122(9):984–91. doi: 10.1289/ehp.1307450 (PMC4154212; doi:10.1289/ehp.1307450)
Supplement: (1.3 MB) PDF [file ehp.1307450.s001.pdf]

Environmental Health Perspectives doi:10.1289/ehp.1307450

Supplemental Material

Pesticide Exposure and Depression among Male Private Pesticide Applicators in the Agricultural Health Study

John D. Beard, David M. Umbach, Jane A. Hoppin, Marie Richards, Michael C.R. Alavanja, Aaron Blair, Dale P. Sandler, and Freya Kamel

Table of Contents **Page**

Figure S1. Directed acyclic graph to identify potential confounders that needed to be controlled to estimate the total effect of pesticide use on self-reported depression among male private pesticide applicators in the Agricultural Health Study 3

Methods, statistical analyses 4

Exposure/confounding weights 4

Missing covariate (diabetes and education) data weights 4

Missing farmer questionnaire weights (if appropriate) 5

Selection/drop out weights 5

Table S1. More characteristics of male private pesticide applicators in the Agricultural Health Study 6

Table S2. Summary statistics for the truncated (at the 1st and 99th percentiles) overall stabilized inverse probability weights[a] for models in Table 2 9

Table S3. Summary statistics for the truncated (at the 1st and 99th percentiles) overall stabilized inverse probability weights[a] for models in Table 3 10

Table S4. Summary statistics for the truncated (at the 1st and 99th percentiles) overall stabilized inverse probability weights[a] for models in Table S5 13

Table S5. Cumulative lifetime days of use of individual pesticides and self-reported depression among male private pesticide applicators in the Agricultural Health Study 19

Table S6. Pesticide use and self-reported depression among male private pesticide applicators in the Agricultural Health Study without weighting for potential biases from missing covariate data, missing farmer questionnaire, or drop out 32

Table S7. Ever-use of pesticide classes and specific pesticides and self-reported depression among male private pesticide applicators in the Agricultural Health Study without weighting for potential biases from missing covariate data, missing farmer questionnaire, or drop out 33

References 35

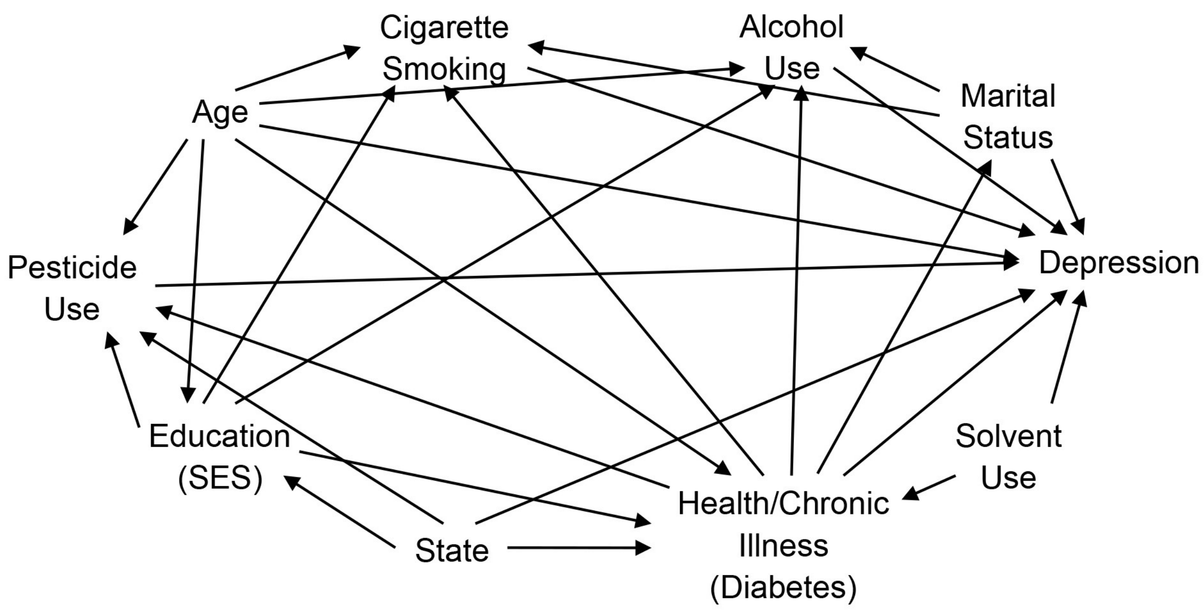

Figure S1. Directed acyclic graph to identify potential confounders that needed to be controlled to estimate the total effect of pesticide use on self-reported depression among male private pesticide applicators in the Agricultural Health Study. Note that switching the direction of the arrows from "Health/Chronic Illness (Diabetes)" to "Cigarette Smoking", from "Health/Chronic Illness (Diabetes)" to "Alcohol Use", and from "Health/Chronic Illness (Diabetes)" to "Marital Status" or adding an arrow from "Age" to "Marital Status" all result in the same two minimally sufficient adjustment sets as the directed acyclic graph shown here.

Methods, statistical analyses

We used standard formulas (Muller and Fetterman 2002; Stokes et al. 2000) to estimate linear, logistic, or ordinal logistic regression models to calculate the various stabilized weights (Hernán et al. 2000; Robins et al. 2000). In all models, the quanitities of interest were the predicted probabilities of exposure, not missing covariate data, completing the farmer questionnaire, or not dropping out of the cohort. The populations to which the weights applied, types of models used, outcome of each model, and covariates included in each model were as follows:

Exposure/confounding weights

Population: 45,827 male applicators not missing data on depression at enrollment and at follow-up and not missing covariate data (for analyses involving information from the farmer questionnaire, this number was 20,471)

Model type: linear, logistic, or ordinal logistic (depending on the nature of the exposure variable)
Outcome: exposure (continuous, dichotomous, or ordered categories)
Covariates in models used to estimate numerators: intercept only
Covariates in models used to estimate denominators: age, diabetes, education, state

Missing covariate (diabetes and education) data weights

Population: 49,142 male applicators not missing data on depression at enrollment and at follow-up (for analyses involving information from the farmer questionnaire, this numbers was 22,300)

Model type: logistic
Outcome: not missing covariate data (dichotomous)
Covariates in models used to estimate numerators: exposure
Covariates in models used to estimate denominators: age, state, exposure, age*exposure, state*exposure

Missing farmer questionnaire weights (if appropriate)

Population: 45,827 male applicators not missing data on depression at enrollment and at follow-up and not missing covariate data

Model type: logistic
Outcome: completed farmer questionnaire (dichotomous)
Covariates in models used to estimate numerators: intercept only
Covariates in models used to estimate denominators: age, diabetes, education, state

Selection/drop out weights

Population: 45,827 male applicators not missing data on depression at enrollment and at follow-up and not missing covariate data (for analyses involving information from the farmer questionnaire, this number was 20,471)

Model type: logistic
Outcome: did not drop out of cohort (dichotomous)
Covariates in models used to estimate numerators: exposure
Covariates in models used to estimate denominators: age, diabetes, education, state, exposure, age*exposure, diabetes*exposure, education*exposure, state*exposure

For more detailed information on inverse probability weighting, see Cole and Hernán (2008), Hernán et al. (2000, 2004), Hernán and Robins (2006), Robins (1998), Robins et al. (2000), and Sato and Matsuyama (2003).

Table S1. More characteristics of male private pesticide applicators in the Agricultural Health Study.

Characteristic	Non-cases [*n* (%)]	PRE-E[a] Cases [*n* (%)]	PRE-E[a] Adjusted OR[b] (95% CI)	PRE-B[a] Cases [*n* (%)]	PRE-B[a] Adjusted OR[b] (95% CI)	POST[a] Cases [*n* (%)]	POST[a] Adjusted OR[b] (95% CI)	*p* for difference among ORs[c]
**Total**	19,506 (100)	474 (100)		540 (100)		688 (100)		
**Marital status**								
Married or living as married	17,121 (88)	402 (85)	Reference	453 (84)	Reference	620 (90)	Reference	
Divorced, separated, or widowed	688 (4)	39 (8)	2.5 (1.8, 3.5)	47 (9)	2.7 (2.0, 3.6)	22 (3)	0.9 (0.6, 1.3)	
Never married	1,676 (9)	33 (7)	1.3 (0.9, 2.0)	38 (7)	1.3 (0.9, 1.9)	46 (7)	0.7 (0.5, 1.0)	< 0.01
Missing	21	0		2		0		
**Number of children in family**								
0	2,787 (14)	53 (11)	Reference	67 (12)	Reference	86 (13)	Reference	
1-3	12,659 (65)	309 (66)	0.9 (0.7, 1.2)	364 (68)	0.8 (0.6, 1.1)	478 (70)	1.2 (1.0, 1.6)	
4-5	3,248 (17)	88 (19)	0.9 (0.6, 1.3)	83 (15)	0.7 (0.5, 0.9)	106 (15)	1.2 (0.9, 1.6)	
≥ 5	702 (4)	18 (4)	0.8 (0.4, 1.4)	23 (4)	0.8 (0.5, 1.4)	17 (2)	1.0 (0.6, 1.7)	0.26
Missing	110	6		3		1		
**Frequency of alcohol consumption during past year (times a week)**								
Never	6,448 (33)	172 (36)	Reference	189 (35)	Reference	219 (32)	Reference	
< 1	6,628 (34)	162 (34)	1.0 (0.8, 1.2)	194 (36)	1.0 (0.8, 1.3)	245 (36)	1.1 (0.9, 1.3)	
1-4	5,281 (27)	105 (22)	0.8 (0.6, 1.1)	132 (25)	0.9 (0.7, 1.1)	171 (25)	0.9 (0.7, 1.1)	
> 4	1,073 (6)	34 (7)	1.2 (0.8, 1.8)	22 (4)	0.7 (0.4, 1.1)	45 (7)	1.2 (0.9, 1.7)	0.45
Missing	76	1		3		8		
**Cigarette smoking status**								
Never	11,155 (57)	230 (49)	Reference	268 (50)	Reference	336 (49)	Reference	
Past	5,935 (31)	175 (37)	1.2 (1.0, 1.5)	201 (37)	1.3 (1.0, 1.5)	236 (35)	1.5 (1.2, 1.7)	
Current	2,342 (12)	66 (14)	1.3 (1.0, 1.8)	69 (13)	1.2 (0.9, 1.6)	112 (16)	1.6 (1.2, 1.9)	0.50
Missing	74	3		2		4		
**Times visited medical doctor or medical assistant about health concern in past year[d]**								
0	3,887 (36)	49 (16)	Reference	43 (14)	Reference	90 (24)	Reference	
1	3,556 (33)	72 (24)	1.6 (1.1, 2.3)	75 (24)	1.9 (1.3, 2.8)	125 (34)	1.6 (1.2, 2.1)	
> 1	3,417 (31)	183 (60)	4.1 (3.0, 5.7)	189 (62)	5.3 (3.8, 7.5)	154 (42)	2.2 (1.7, 2.9)	< 0.01
Missing	130	2		8		2		

Characteristic	Non-cases [*n* (%)]	PRE-E[a] Cases [*n* (%)]	PRE-E[a] Adjusted OR[b] (95% CI)	PRE-B[a] Cases [*n* (%)]	PRE-B[a] Adjusted OR[b] (95% CI)	POST[a] Cases [*n* (%)]	POST[a] Adjusted OR[b] (95% CI)	*p* for difference among ORs[c]
**Years lived or worked on farm over lifetime[d]**								
< 21	967 (9)	17 (6)	0.8 (0.5, 1.3)	27 (9)	1.3 (0.8, 2.0)	41 (11)	1.4 (0.9, 1.9)	
21-30	1,387 (13)	29 (10)	1.0 (0.6, 1.4)	39 (13)	1.3 (1.0, 1.8)	58 (16)	1.4 (1.0, 1.9)	
> 30	8,466 (78)	258 (85)	Reference	243 (79)	Reference	265 (73)	Reference	0.32
Missing	170	2		6		7		
**Size of farm worked last year (acres)**								
Didn't work on a farm or none	591 (3)	16 (3)	Reference	21 (4)	Reference	22 (3)	Reference	
< 50	2,415 (13)	72 (16)	1.1 (0.7, 2.0)	77 (15)	0.9 (0.6, 1.5)	81 (13)	0.8 (0.5, 1.4)	
50-499	8,949 (48)	228 (50)	0.9 (0.5, 1.6)	260 (50)	0.7 (0.4, 1.2)	301 (47)	0.9 (0.6, 1.4)	
> 499	6,781 (36)	144 (31)	0.8 (0.5, 1.4)	163 (31)	0.6 (0.4, 1.0)	238 (37)	0.9 (0.6, 1.5)	0.26
Missing	770	14		19		46		
**Wear chemical resistant gloves when personally handle pesticides**								
No	5,156 (26)	129 (27)	Reference	147 (27)	Reference	198 (29)	Reference	
Yes	14,350 (74)	345 (73)	1.0 (0.8, 1.2)	393 (73)	0.9 (0.8, 1.1)	490 (71)	0.9 (0.7, 1.0)	0.68
**Ever have job off farm[d]**								
No	3,725 (34)	91 (30)	Reference	100 (32)	Reference	110 (30)	Reference	
Yes	7,151 (66)	215 (70)	1.2 (1.0, 1.6)	211 (68)	1.1 (0.9, 1.4)	259 (70)	1.2 (0.9, 1.5)	0.83
Missing	114	0		4		2		
**Exposed to solvents (other than gasoline) at longest held non-farm job[d]**								
No	8,961 (82)	235 (77)	Reference	247 (79)	Reference	311 (84)	Reference	
Yes	1,915 (18)	71 (23)	1.5 (1.1, 1.9)	64 (21)	1.2 (0.9, 1.6)	58 (16)	0.8 (0.6, 1.1)	0.01
Missing	114	0		4		2		

Abbreviations: CI, confidence interval; OR, odds ratio; POST, post-enrollment; PRE-B, pre-enrollment both; PRE-E, pre-enrollment enrollment only.

[a]Cases divided into three groups based on when the physician-diagnosis of depression occurred (before or after enrollment) and on when it was reported via the AHS contacts (at enrollment, at follow-up, or both). The PRE-E group included applicators who reported a previous diagnosis of

depression at enrollment, but who did not confirm their pre-enrollment diagnosis at follow-up. The PRE-B group included applicators who reported a previous diagnosis of depression at both enrollment and follow-up, or who reported a previous diagnosis at follow-up only but with an age at diagnosis less than their age at enrollment. The POST group included applicators who reported a previous diagnosis of depression at follow-up but not enrollment, and whose reported age at diagnosis equaled or exceeded their age at enrollment. [b]Adjusted for age at enrollment (modeled with a cubic polynomial) and state of residence. [c]Differences among case-group-specific ORs tested via Wald χ^2 tests. [d]Data available only for 11,982 applicators who completed the farmer questionnaire.

Table S2. Summary statistics for the truncated (at the 1st and 99th percentiles) overall stabilized inverse probability weights[a] for models in Table 2.

Variable	Mean	Standard Deviation	1st Percentile	5th Percentile	Median	95th Percentile	99th Percentile
Cumulative lifetime days personally mixed or applied pesticides							
Categorical[b]	1.05	0.32	0.58	0.67	0.97	1.68	2.27
Trend[c]	1.00	0.23	0.67	0.73	0.95	1.46	1.81
Ever diagnosed with pesticide poisoning[d]	0.99	0.34	0.52	0.57	0.95	1.61	2.42
Ever experienced an incident of unusually high personal pesticide exposure[d]	1.05	0.32	0.58	0.67	0.97	1.68	2.27

[a]Weights adjusted for age at enrollment (modeled with a restricted, quadratic spline with knots at ages 40, 48, and 57 years based on percentiles of the age distribution in cases), ever diagnosed with diabetes, education level, state of residence, not missing covariate data (conditional on age, state, the exposure, and pairwise interaction terms between each covariate and the exposure), and not dropping out of the AHS cohort (conditional on age, diabetes, education, state, the exposure, and pairwise interaction terms between each covariate and the exposure). [b]Category boundaries set at quartiles of cumulative lifetime days of pesticide use among all male private pesticide applicators. [c]Used within-category medians. [d]Data available only for 11,982 applicators who completed the farmer questionnaire. Weights additionally adjusted for completing the farmer questionnaire (conditional on age, diabetes, education, and state).

Table S3. Summary statistics for the truncated (at the 1st and 99th percentiles) overall stabilized inverse probability weights[a] for models in Table 3.

Ever personally mixed or applied	Mean	Standard deviation	1st Percentile	5th Percentile	Median	95th Percentile	99th Percentile
Fumigants	0.99	0.50	0.34	0.42	0.81	2.00	2.70
Aluminum phosphide	0.99	0.21	0.60	0.75	0.96	1.41	1.75
Carbon tetrachloride/carbon disulfide (80/20 mix)	0.99	0.23	0.33	0.73	0.95	1.41	1.91
Ethylene dibromide	0.99	0.23	0.44	0.73	0.95	1.46	1.84
Methyl bromide	0.98	0.70	0.27	0.31	0.79	2.06	5.70
Fungicides	1.00	0.59	0.41	0.48	0.77	2.48	2.87
Benomyl[b]	0.99	0.41	0.25	0.42	0.88	1.63	3.28
Captan	0.99	0.22	0.56	0.72	0.96	1.43	1.85
Chlorothalonil	0.98	0.35	0.30	0.51	0.90	1.63	2.57
Maneb/mancozeb	0.98	0.41	0.24	0.36	0.88	1.63	3.07
Metalaxyl	0.99	0.54	0.35	0.41	0.79	2.18	2.75
Ziram	1.00	0.21	0.66	0.74	0.95	1.42	1.73
Herbicides	0.99	0.24	0.64	0.72	0.94	1.47	1.90
Alachlor	0.99	0.28	0.62	0.64	0.96	1.52	2.28
Butylate	0.98	0.30	0.53	0.57	0.97	1.44	2.60
Chlorimuron-ethyl	0.99	0.28	0.61	0.66	0.92	1.54	2.18
Dicamba	0.98	0.69	0.47	0.51	0.73	2.06	4.82
EPTC	0.98	0.38	0.51	0.55	0.94	1.33	3.60
Glyphosate	0.99	0.32	0.59	0.62	0.93	1.63	2.40
Imazethapyr	0.98	0.79	0.48	0.55	0.76	1.72	6.17
Metolachlor	0.99	0.35	0.61	0.62	0.91	1.63	2.59
Paraquat	0.99	0.43	0.36	0.47	0.85	1.85	2.38
Pendimethalin	1.00	0.22	0.63	0.73	0.96	1.44	1.79
Petroleum oil	0.99	0.28	0.62	0.63	0.96	1.47	2.34
Trifluralin	0.99	0.43	0.56	0.58	0.85	1.80	3.08
Phenoxy herbicides	0.99	0.34	0.58	0.61	0.90	1.70	2.34
2,4-D	0.99	0.36	0.56	0.60	0.88	1.75	2.44
2,4,5-T	0.98	0.35	0.39	0.55	0.92	1.58	2.86
2,4,5-TP	0.99	0.23	0.44	0.72	0.95	1.41	1.94

Ever personally mixed or applied	Mean	Standard deviation	1st Percentile	5th Percentile	Median	95th Percentile	99th Percentile
Triazine herbicides	0.99	0.40	0.45	0.58	0.85	1.85	2.45
Atrazine	0.99	0.37	0.47	0.64	0.86	1.71	2.46
Cyanazine	0.98	0.59	0.51	0.55	0.80	1.52	4.33
Metribuzin	0.98	0.45	0.54	0.55	0.86	1.68	3.31
Insecticides	1.00	0.24	0.63	0.72	0.94	1.48	1.90
Carbamates[b]	1.00	0.56	0.57	0.62	0.82	1.99	4.05
Aldicarb	0.99	0.46	0.29	0.40	0.88	1.77	3.38
Carbaryl	0.99	0.56	0.52	0.59	0.76	2.28	3.58
Carbofuran	0.99	0.26	0.57	0.65	0.93	1.47	2.18
Organochlorine insecticides	0.98	0.61	0.50	0.54	0.73	2.24	4.00
Aldrin	0.95	0.42	0.31	0.39	0.90	1.58	3.33
Chlordane	0.98	0.43	0.37	0.48	0.85	1.80	3.12
DDT	0.96	0.67	0.33	0.38	0.79	2.16	4.77
Dieldrin	0.97	0.26	0.25	0.52	0.95	1.40	2.04
Heptachlor	0.95	0.37	0.28	0.37	0.91	1.54	2.75
Lindane	0.99	0.26	0.48	0.57	0.94	1.40	2.37
Toxaphene	0.98	0.32	0.36	0.51	0.91	1.55	2.43
Organophosphate insecticides	0.99	0.27	0.58	0.70	0.92	1.55	2.03
Chlorpyrifos	0.99	0.23	0.67	0.69	0.95	1.44	1.95
Coumaphos	0.99	0.22	0.59	0.75	0.95	1.42	1.82
Diazinon	1.00	0.39	0.44	0.55	0.85	1.74	2.41
Dichlorvos	0.98	0.24	0.42	0.64	0.94	1.37	2.12
Fonofos	0.98	0.43	0.51	0.56	0.96	1.32	4.05
Malathion	0.99	0.28	0.60	0.67	0.91	1.58	2.13
Parathion	0.99	0.34	0.34	0.54	0.90	1.62	2.40
Phorate	0.99	0.43	0.52	0.58	0.97	1.48	3.59
Terbufos	0.99	0.38	0.59	0.61	0.93	1.68	2.90
Trichlorfon	1.00	0.21	0.66	0.74	0.95	1.43	1.78
Pyrethroid insecticides	0.99	0.27	0.55	0.60	0.95	1.44	2.24
Permethrin (for animals)	0.98	0.28	0.44	0.54	0.95	1.37	2.47
Permethrin (for crops)	0.99	0.24	0.58	0.70	0.95	1.47	1.86

Abbreviations: 2,4-D, (2,4-dichlorophenoxy)acetic acid; 2,4,5-T, (2,4,5-trichlorophenoxy)acetic acid; 2,4,5-TP, (*RS*)-2-(2,4,5-trichlorophenoxy)propionic acid; DDT, 1,1,1-trichloro-2,2-bis(4-chlorophenyl)ethane; EPTC, *S*-ethyl dipropyl(thiocarbamate).

[a]Weights adjusted for age at enrollment (modeled with a restricted, quadratic spline with knots at ages 40, 48, and 57 years based on percentiles of the age distribution in cases), ever diagnosed with diabetes, education level, state of residence, not missing covariate data (conditional on age, state, the exposure, and pairwise interaction terms between each covariate and the exposure), and not dropping out of the AHS cohort (conditional on age, diabetes, education, state, the exposure, and pairwise interaction terms between each covariate and the exposure). [b]Benomyl is also included in carbamates.

Table S4. Summary statistics for the truncated (at the 1st and 99th percentiles) overall stabilized inverse probability weights[a] for models in Table S5.

Cumulative lifetime days personally mixed or applied[b,c]	Mean	Standard deviation	1st Percentile	5th Percentile	Median	95th Percentile	99th Percentile
Fumigants							
Aluminum phosphide[d]							
Categorical[e]	0.99	0.31	0.53	0.63	0.93	1.63	2.12
Trend[f]	1.00	0.31	0.56	0.64	0.92	1.64	2.11
Carbon tetrachloride/carbon disulfide (80/20 mix)[d]							
Categorical	0.99	0.31	0.35	0.65	0.92	1.61	2.13
Trend[f]	1.00	0.31	0.57	0.64	0.93	1.63	2.12
Ethylene dibromide[d]							
Categorical[e]	1.00	0.35	0.50	0.62	0.92	1.72	2.35
Trend[f]	1.00	0.31	0.56	0.64	0.92	1.62	2.13
Methyl bromide							
Categorical	1.02	0.44	0.27	0.63	0.89	1.82	3.41
Trend[f]	1.00	0.21	0.67	0.74	0.95	1.43	1.76
Fungicides							
Benomyl[d,g]							
Categorical[e]	1.00	0.39	0.28	0.60	0.90	1.79	2.48
Trend[f]	1.00	0.31	0.55	0.64	0.92	1.63	2.12
Captan							
Categorical	1.00	0.22	0.58	0.73	0.96	1.41	1.81
Trend[f]	1.00	0.21	0.67	0.74	0.95	1.42	1.75
Chlorothalonil							
Categorical	1.00	0.28	0.44	0.70	0.94	1.56	2.16
Trend[f]	1.00	0.21	0.67	0.74	0.95	1.42	1.75
Maneb/mancozeb[d]							
Categorical	1.00	0.38	0.28	0.60	0.90	1.77	2.42
Trend[f]	1.00	0.31	0.57	0.64	0.92	1.62	2.12

Cumulative lifetime days personally mixed or applied[b,c]	Mean	Standard deviation	1st Percentile	5th Percentile	Median	95th Percentile	99th Percentile
Metalaxyl[d]							
Categorical	1.08	0.58	0.34	0.55	0.90	2.38	3.41
Trend[f]	0.99	0.29	0.56	0.65	0.93	1.59	2.09
Herbicides							
Alachlor							
Categorical	1.00	0.23	0.60	0.67	0.96	1.42	2.01
Trend[f]	1.00	0.22	0.68	0.74	0.95	1.44	1.75
Butylate[d]							
Categorical	0.99	0.29	0.44	0.64	0.97	1.53	2.19
Trend[f]	1.00	0.31	0.57	0.64	0.93	1.63	2.12
Chlorimuron-ethyl[d]							
Categorical	0.99	0.33	0.56	0.63	0.92	1.68	2.24
Trend[f]	1.00	0.31	0.57	0.64	0.92	1.63	2.10
Dicamba							
Categorical	1.07	0.61	0.48	0.53	0.99	1.64	5.07
Trend[f]	1.00	0.21	0.69	0.74	0.96	1.41	1.75
EPTC							
Categorical	0.98	0.22	0.51	0.68	0.96	1.35	1.96
Trend[f]	1.00	0.21	0.67	0.74	0.95	1.42	1.75
Glyphosate							
Categorical	1.05	0.36	0.57	0.66	0.95	1.80	2.38
Trend[f]	1.00	0.22	0.67	0.74	0.95	1.44	1.77
Imazethapyr							
Categorical	1.04	0.60	0.51	0.55	0.94	1.70	5.79
Trend[f]	1.00	0.21	0.67	0.74	0.95	1.42	1.77
Metolachlor							
Categorical	1.00	0.27	0.59	0.66	0.95	1.45	2.29
Trend[f]	1.00	0.22	0.67	0.74	0.95	1.43	1.79
Paraquat[d]							
Categorical	1.04	0.46	0.33	0.58	0.90	2.04	2.74
Trend[f]	1.00	0.31	0.55	0.64	0.92	1.63	2.08

Cumulative lifetime days personally mixed or applied[b,c]	Mean	Standard deviation	1st Percentile	5th Percentile	Median	95th Percentile	99th Percentile
Pendimethalin[d]							
Categorical	1.02	0.36	0.52	0.61	0.94	1.76	2.38
Trend[f]	0.99	0.31	0.57	0.64	0.92	1.63	2.13
Petroleum oil[d]							
Categorical	0.99	0.31	0.54	0.64	0.94	1.60	2.18
Trend[f]	1.00	0.31	0.57	0.63	0.93	1.65	2.14
Trifluralin							
Categorical	1.05	0.32	0.52	0.64	1.02	1.46	2.68
Trend[f]	1.00	0.21	0.68	0.74	0.95	1.42	1.78
Phenoxy herbicides							
2,4-D							
Categorical	1.10	0.37	0.53	0.64	1.04	1.71	2.86
Trend[f]	1.00	0.21	0.68	0.74	0.95	1.42	1.76
2,4,5-T[d]							
Categorical	0.99	0.33	0.33	0.55	0.92	1.63	2.41
Trend[f]	1.00	0.31	0.58	0.63	0.93	1.64	2.13
2,4,5-TP[d]							
Categorical[e]	0.99	0.31	0.50	0.64	0.92	1.63	2.15
Trend[f]	1.00	0.31	0.57	0.63	0.93	1.63	2.13
Triazine herbicides							
Atrazine							
Categorical	1.05	0.28	0.54	0.66	1.02	1.51	2.26
Trend[f]	1.00	0.22	0.68	0.74	0.95	1.45	1.76
Cyanazine							
Categorical	1.02	0.40	0.51	0.58	0.98	1.41	3.65
Trend[f]	1.00	0.21	0.68	0.74	0.95	1.42	1.85
Metribuzin[d]							
Categorical	1.00	0.41	0.41	0.51	0.94	1.51	3.44
Trend[f]	1.00	0.30	0.57	0.63	0.93	1.62	2.08

Cumulative lifetime days personally mixed or applied[b,c]	Mean	Standard deviation	1st Percentile	5th Percentile	Median	95th Percentile	99th Percentile
Insecticides							
Carbamates[g]							
Aldicarb[d]							
Categorical[e]	1.00	0.43	0.33	0.59	0.89	1.88	2.92
Trend[f]	0.99	0.30	0.56	0.64	0.92	1.61	2.08
Carbaryl[d]							
Categorical	1.28	0.89	0.44	0.56	0.87	3.25	4.59
Trend[f]	1.00	0.31	0.55	0.64	0.92	1.63	2.15
Carbofuran							
Categorical	1.00	0.22	0.60	0.72	0.95	1.43	1.91
Trend[f]	1.00	0.21	0.68	0.74	0.95	1.42	1.74
Organochlorine insecticides							
Aldrin[d]							
Categorical	0.98	0.32	0.24	0.41	0.93	1.55	2.36
Trend[f]	1.00	0.30	0.57	0.64	0.92	1.62	2.08
Chlordane[d]							
Categorical	0.99	0.40	0.28	0.53	0.88	1.73	2.79
Trend[f]	1.00	0.31	0.56	0.64	0.93	1.63	2.09
DDT[d]							
Categorical	0.99	0.43	0.27	0.43	0.88	1.80	3.03
Trend[f]	1.00	0.31	0.56	0.64	0.93	1.65	2.12
Dieldrin[d]							
Categorical[e]	0.99	0.31	0.28	0.65	0.92	1.61	2.12
Trend[f]	1.00	0.31	0.57	0.63	0.93	1.64	2.11
Heptachlor[d]							
Categorical	0.98	0.32	0.25	0.56	0.92	1.57	2.26
Trend[f]	1.00	0.30	0.57	0.64	0.92	1.62	2.09
Lindane[d]							
Categorical	0.99	0.30	0.39	0.66	0.93	1.60	2.18
Trend[f]	1.00	0.31	0.56	0.64	0.92	1.63	2.09

Cumulative lifetime days personally mixed or applied[b,c]	Mean	Standard deviation	1st Percentile	5th Percentile	Median	95th Percentile	99th Percentile
Toxaphene[d]							
Categorical	1.00	0.34	0.47	0.64	0.91	1.66	2.26
Trend[f]	1.00	0.31	0.55	0.63	0.93	1.63	2.12
Organophosphate insecticides							
Chlorpyrifos							
Categorical	1.00	0.22	0.66	0.73	0.95	1.45	1.84
Trend[f]	1.00	0.22	0.67	0.73	0.95	1.44	1.80
Coumaphos							
Categorical	1.00	0.21	0.65	0.75	0.96	1.41	1.76
Trend[f]	1.00	0.21	0.67	0.74	0.95	1.42	1.75
Diazinon[d]							
Categorical	1.02	0.39	0.37	0.62	0.91	1.80	2.44
Trend[f]	1.00	0.31	0.56	0.63	0.92	1.62	2.11
Dichlorvos							
Categorical	0.99	0.20	0.44	0.76	0.95	1.37	1.81
Trend[f]	1.00	0.21	0.67	0.74	0.95	1.42	1.75
Fonofos							
Categorical	0.98	0.22	0.53	0.65	0.96	1.34	1.90
Trend[f]	1.00	0.21	0.68	0.74	0.96	1.42	1.78
Malathion[d]							
Categorical	1.02	0.33	0.49	0.64	0.94	1.65	2.31
Trend[f]	1.00	0.31	0.56	0.64	0.92	1.63	2.10
Parathion[d]							
Categorical	1.00	0.35	0.41	0.62	0.91	1.72	2.31
Trend[f]	1.00	0.31	0.56	0.63	0.92	1.63	2.11
Phorate[d]							
Categorical	0.97	0.28	0.46	0.55	0.93	1.46	2.15
Trend[f]	1.00	0.31	0.57	0.64	0.92	1.62	2.15
Terbufos							
Categorical	0.99	0.23	0.59	0.67	0.97	1.36	2.06
Trend[f]	1.00	0.21	0.68	0.74	0.95	1.42	1.78

Cumulative lifetime days personally mixed or applied[b,c]	Mean	Standard deviation	1st Percentile	5th Percentile	Median	95th Percentile	99th Percentile
Pyrethroid insecticides							
Permethrin (for animals)							
Categorical	0.99	0.22	0.45	0.73	0.96	1.37	1.82
Trend[f]	1.00	0.21	0.68	0.74	0.96	1.41	1.72
Permethrin (for crops)							
Categorical	1.00	0.24	0.65	0.73	0.96	1.48	1.89
Trend[f]	1.00	0.21	0.66	0.74	0.96	1.42	1.76

Abbreviations: 2,4-D, (2,4-dichlorophenoxy)acetic acid; 2,4,5-T, (2,4,5-trichlorophenoxy)acetic acid; 2,4,5-TP, (*RS*)-2-(2,4,5-trichlorophenoxy)propionic acid; DDT, 1,1,1-trichloro-2,2-bis(4-chlorophenyl)ethane; EPTC, *S*-ethyl dipropyl(thiocarbamate); POST, post-enrollment; PRE-B, pre-enrollment both; PRE-E, pre-enrollment enrollment only.

[a]Weights adjusted for age at enrollment (modeled with a restricted, quadratic spline with knots at ages 40, 48, and 57 years based on percentiles of the age distribution in cases), ever diagnosed with diabetes, education level, state of residence, not missing covariate data (conditional on age, state, the exposure, and pairwise interaction terms between each covariate and the exposure), and not dropping out of the AHS cohort (conditional on age, diabetes, education, state, the exposure, and pairwise interaction terms between each covariate and the exposure). [b]Category boundaries set at tertiles of cumulative lifetime days of use for each pesticide among all male private pesticide applicators who used it. [c]Fewer than five PRE-E, PRE-B, or POST cases (see footnote a at the bottom of Table S1 for a description of the three case groups) used trichlorfon or ziram at every exposure level. [d]Data available only for 11,982 applicators who completed the farmer questionnaire. Weights additionally adjusted for completing the farmer questionnaire (conditional on age, diabetes, education, and state). [e]Category boundaries set at the median of cumulative lifetime days of use for each pesticide among all male private pesticide applicators who used it. [f]Used within-category medians. [g]Benomyl is also included in carbamates.

Table S5. Cumulative lifetime days of use of individual pesticides and self-reported depression among male private pesticide applicators in the Agricultural Health Study.

Cumulative lifetime days personally mixed or applied[a,b]	Non-cases [*n* (%)]	PRE-E[c] Cases [*n* (%)]	PRE-E[c] IP-weighted OR[d] (95% CI)	PRE-B[c] Cases [*n* (%)]	PRE-B[c] IP-weighted OR[d] (95% CI)	POST[c] Cases [*n* (%)]	POST[c] IP-weighted OR[d] (95% CI)	*p* for difference among ORs[e]
Total	19,506 (100)	474 (100)		540 (100)		688 (100)		
Fumigants								
Aluminum phosphide[f,g]								
0 (Median = 0.0)	10,348 (96)	284 (95)	Reference	284 (94)	Reference	345 (94)	Reference	
1-8 (3.5)	217 (2)	6 (2)	0.8 (0.3, 1.9)	11 (4)	1.6 (0.8, 3.2)	13 (4)	1.6 (0.8, 3.1)	
> 8 (14.5)	168 (2)	9 (3)	1.9 (0.9, 3.8)	6 (2)	1.0 (0.4, 2.3)	8 (2)	1.6 (0.7, 3.5)	0.48
Missing	257	7		14		5		
Trend (IQR = 8.8)[h]			1.4 (0.9, 2.2)		1.1 (0.7, 1.7)		1.4 (0.9, 2.2)	0.58
Carbon tetrachloride/carbon disulfide (80/20 mix)[f,g]								
0 (Median = 0.0)	10,210 (95)	277 (93)	Reference	284 (95)	Reference	343 (94)	Reference	
1-12 (3.5)	378 (4)	15 (5)	1.7 (0.9, 3.2)	12 (4)	1.3 (0.6, 2.4)	17 (5)	1.2 (0.6, 2.2)	
> 12 (54.3)	129 (1)	7 (2)	2.1 (1.0, 4.7)	3 (1)	[i]	6 (2)	1.2 (0.5, 2.7)	0.65
Missing	273	7		16		5		
Trend (IQR = 21.0)[h]			1.4 (1.0, 1.8)		1.0 (0.6, 1.6)		1.1 (0.8, 1.5)	0.41
Ethylene dibromide[f]								
0 (0.0)	10,294 (96)	280 (94)	Reference	285 (94)	Reference	349 (95)	Reference	
1-8 (3.5)	151 (1)	6 (2)	1.3 (0.5, 3.8)	6 (2)	1.7 (0.6, 4.9)	6 (2)	1.0 (0.4, 2.8)	
9-28 (15.5)	174 (2)	6 (2)	1.4 (0.6, 3.4)	4 (1)	[i]	6 (2)	1.0 (0.4, 2.4)	
> 28 (87.5)	107 (1)	7 (2)	2.6 (1.2, 5.8)	7 (2)	2.7 (1.2, 6.1)	5 (1)	1.7 (0.6, 4.5)	0.94
Missing	264	7		13		5		
Trend (46.3)[h]			1.7 (1.1, 2.5)		1.7 (1.1, 2.6)		1.3 (0.8, 2.2)	0.69
Methyl bromide								
0 (0.0)	16,516 (86)	392 (84)	Reference	446 (84)	Reference	568 (84)	Reference	
1-12 (8.0)	1,008 (5)	20 (4)	1.0 (0.5, 2.1)	29 (5)	1.2 (0.6, 2.1)	43 (6)	1.5 (1.0, 2.4)	
13-54 (28.0)	1,221 (6)	36 (8)	1.3 (0.9, 1.8)	35 (7)	1.1 (0.8, 1.6)	35 (5)	0.8 (0.6, 1.2)	
> 54 (122.5)	512 (3)	18 (4)	1.6 (1.0, 2.6)	20 (4)	1.4 (0.9, 2.2)	27 (4)	1.6 (1.1, 2.4)	0.59

Cumulative lifetime days personally mixed or applied[a,b]	Non-cases [*n* (%)]	PRE-E[c] Cases [*n* (%)]	PRE-E[c] IP-weighted OR[d] (95% CI)	PRE-B[c] Cases [*n* (%)]	PRE-B[c] IP-weighted OR[d] (95% CI)	POST[c] Cases [*n* (%)]	POST[c] IP-weighted OR[d] (95% CI)	*p* for difference among ORs[e]
Missing	249	8		10		15		
Trend (46.3)[h]			1.2 (1.0, 1.4)		1.1 (1.0, 1.4)		1.2 (1.0, 1.4)	0.88
Fungicides								
Benomyl[f,g,j]								
0 (0.0)	9,925 (93)	272 (92)	Reference	272 (92)	Reference	333 (92)	Reference	
1-25 (12.3)	435 (4)	15 (5)	1.3 (0.7, 2.7)	13 (4)	1.5 (0.8, 3.1)	17 (5)	0.7 (0.4, 1.4)	
> 25 (103.3)	313 (3)	9 (3)	1.1 (0.5, 2.2)	12 (4)	1.4 (0.8, 2.7)	14 (4)	1.5 (0.8, 2.7)	0.50
Missing	317	10		18		7		
Trend (52.5)[h]			1.0 (0.7, 1.5)		1.2 (0.9, 1.6)		1.2 (0.9, 1.7)	0.72
Captan								
0 (0.0)	16,187 (89)	385 (88)	Reference	415 (85)	Reference	552 (89)	Reference	
> 0-0.25 (0.3)	1,194 (7)	31 (7)	1.1 (0.7, 1.7)	50 (10)	1.6 (1.2, 2.2)	32 (5)	0.8 (0.5, 1.1)	
> 0.25-8 (3.5)	187 (1)	2 (< 1)	[i]	7 (1)	1.4 (0.6, 3.0)	9 (1)	1.5 (0.7, 2.9)	
> 8 (64.0)	590 (3)	20 (5)	1.4 (0.9, 2.2)	19 (4)	1.3 (0.8, 2.1)	29 (5)	1.5 (1.0, 2.3)	0.06
Missing	1,348	36		49		66		
Trend (12.5)[h]			1.1 (1.0, 1.2)		1.0 (1.0, 1.1)		1.1 (1.0, 1.2)	0.75
Chlorothalonil								
0 (0.0)	18,036 (94)	437 (94)	Reference	490 (93)	Reference	622 (92)	Reference	
1-12 (8.0)	427 (2)	10 (2)	0.9 (0.4, 2.0)	19 (4)	1.7 (0.9, 3.1)	19 (3)	1.2 (0.6, 2.1)	
13-88 (54.3)	419 (2)	10 (2)	1.2 (0.6, 2.4)	13 (2)	1.2 (0.7, 2.1)	14 (2)	1.2 (0.7, 2.2)	
> 88 (200.0)	394 (2)	8 (2)	0.8 (0.4, 1.7)	6 (1)	0.6 (0.3, 1.5)	18 (3)	1.3 (0.8, 2.1)	0.67
Missing	230	9		12		15		
Trend (111.8)[h]			0.9 (0.6, 1.3)		0.8 (0.6, 1.2)		1.2 (0.9, 1.5)	0.32
Maneb/mancozeb[f]								
0 (0.0)	9,896 (93)	268 (91)	Reference	273 (92)	Reference	330 (90)	Reference	
1-12 (7.0)	361 (3)	11 (4)	1.4 (0.6, 3.1)	4 (1)	[i]	14 (4)	1.1 (0.5, 2.3)	
13-56 (50.8)	238 (2)	10 (3)	2.0 (1.0, 3.9)	7 (2)	1.0 (0.5, 2.3)	13 (4)	1.7 (0.9, 3.0)	
> 56 (224.8)	197 (2)	5 (2)	0.9 (0.4, 2.3)	14 (5)	3.0 (1.7, 5.3)	8 (2)	1.0 (0.5, 2.2)	0.12
Missing	298	12		17		6		
Trend (96.3)[h]			1.0 (0.8, 1.4)		1.6 (1.2, 2.0)		1.1 (0.8, 1.4)	0.04

Cumulative lifetime days personally mixed or applied[a,b]	Non-cases [*n* (%)]	PRE-E[c] Cases [*n* (%)]	PRE-E[c] IP-weighted OR[d] (95% CI)	PRE-B[c] Cases [*n* (%)]	PRE-B[c] IP-weighted OR[d] (95% CI)	POST[c] Cases [*n* (%)]	POST[c] IP-weighted OR[d] (95% CI)	*p* for difference among ORs[e]
Metalaxyl[f]								
0 (0.0)	8,790 (83)	238 (81)	Reference	242 (81)	Reference	304 (85)	Reference	
1-12 (3.5)	1,015 (10)	32 (11)	1.0 (0.6, 1.7)	29 (10)	1.4 (0.9, 2.2)	30 (8)	0.7 (0.5, 1.1)	
13-28 (28.0)	353 (3)	10 (3)	0.9 (0.5, 1.8)	8 (3)	0.8 (0.4, 1.8)	12 (3)	0.8 (0.4, 1.6)	
> 28 (56.0)	486 (5)	14 (5)	1.1 (0.6, 2.0)	20 (7)	1.4 (0.9, 2.3)	10 (3)	0.7 (0.3, 1.3)	0.31
Missing	346	12		16		15		
Trend (50.8)[h]			1.1 (0.7, 1.8)		1.3 (0.8, 2.0)		0.7 (0.4, 1.2)	0.20
Herbicides								
Alachlor								
0 (0.0)	8,201 (45)	172 (38)	Reference	197 (38)	Reference	272 (42)	Reference	
1-25 (8.8)	4,619 (25)	121 (27)	1.3 (1.0, 1.6)	137 (27)	1.2 (0.9, 1.5)	179 (28)	1.2 (1.0, 1.5)	
26-109 (56.0)	3,167 (17)	94 (21)	1.5 (1.1, 1.9)	108 (21)	1.4 (1.1, 1.8)	110 (17)	1.0 (0.8, 1.3)	
> 109 (224.8)	2,440 (13)	68 (15)	1.4 (1.0, 1.8)	72 (14)	1.2 (0.9, 1.6)	86 (13)	1.1 (0.9, 1.4)	0.42
Missing	1,079	19		26		41		
Trend (96.0)[h]			1.1 (1.0, 1.2)		1.1 (1.0, 1.2)		1.0 (0.9, 1.1)	0.53
Butylate[f]								
0 (0.0)	7,616 (71)	207 (71)	Reference	200 (66)	Reference	263 (73)	Reference	
1-9 (8.8)	1,080 (10)	32 (11)	1.2 (0.8, 1.8)	37 (12)	1.0 (0.7, 1.5)	30 (8)	0.7 (0.4, 1.0)	
10-51 (24.5)	1,147 (11)	28 (10)	0.9 (0.6, 1.4)	37 (12)	1.2 (0.8, 1.8)	45 (12)	1.1 (0.8, 1.6)	
> 51 (108.5)	846 (8)	24 (8)	1.1 (0.7, 1.7)	28 (9)	1.2 (0.8, 1.8)	24 (7)	0.8 (0.5, 1.3)	0.27
Missing	301	15		13		9		
Trend (47.3)[h]			1.0 (0.8, 1.3)		1.1 (0.9, 1.3)		0.9 (0.8, 1.1)	0.46
Chlorimuron-ethyl[f]								
0 (0.0)	7,240 (68)	210 (71)	Reference	185 (61)	Reference	249 (68)	Reference	
1-9 (8.8)	2,264 (21)	51 (17)	0.7 (0.5, 1.0)	82 (27)	1.4 (1.1, 1.9)	76 (21)	1.0 (0.7, 1.3)	
10-25 (24.5)	673 (6)	16 (5)	0.8 (0.5, 1.4)	21 (7)	1.2 (0.8, 2.0)	26 (7)	1.2 (0.7, 1.8)	
> 25 (56.0)	530 (5)	20 (7)	1.3 (0.8, 2.1)	13 (4)	0.9 (0.5, 1.6)	14 (4)	0.7 (0.4, 1.3)	0.03
Missing	283	9		14		6		
Trend (15.8)[h]			1.0 (0.9, 1.2)		1.0 (0.9, 1.1)		0.9 (0.8, 1.1)	0.64

Cumulative lifetime days personally mixed or applied[a,b]	Non-cases [*n* (%)]	PRE-E[c] Cases [*n* (%)]	PRE-E[c] IP-weighted OR[d] (95% CI)	PRE-B[c] Cases [*n* (%)]	PRE-B[c] IP-weighted OR[d] (95% CI)	POST[c] Cases [*n* (%)]	POST[c] IP-weighted OR[d] (95% CI)	*p* for difference among ORs[e]
Dicamba								
0 (0.0)	8,367 (45)	209 (46)	Reference	217 (43)	Reference	280 (44)	Reference	
1-20 (8.8)	4,315 (23)	93 (20)	0.8 (0.6, 1.1)	134 (27)	1.0 (0.8, 1.3)	146 (23)	0.9 (0.7, 1.2)	
21-56 (38.8)	3,010 (16)	74 (16)	1.0 (0.7, 1.3)	77 (15)	1.0 (0.8, 1.3)	100 (16)	1.0 (0.8, 1.3)	
> 56 (116.0)	2,715 (15)	78 (17)	1.1 (0.9, 1.5)	77 (15)	1.1 (0.9, 1.5)	107 (17)	1.2 (1.0, 1.5)	0.94
Missing	1,099	20		35		55		
Trend (99.8)[h]			1.1 (0.9, 1.4)		1.1 (0.9, 1.3)		1.1 (0.9, 1.4)	0.93
EPTC								
0 (0.0)	14,440 (79)	335 (75)	Reference	397 (80)	Reference	492 (77)	Reference	
1-9 (8.8)	1,800 (10)	50 (11)	1.2 (0.9, 1.7)	47 (9)	1.0 (0.7, 1.4)	77 (12)	1.2 (0.9, 1.6)	
10-25 (24.5)	920 (5)	24 (5)	1.1 (0.7, 1.7)	26 (5)	1.1 (0.7, 1.7)	38 (6)	1.2 (0.8, 1.7)	
> 25 (103.3)	1,212 (7)	38 (9)	1.4 (1.0, 1.9)	28 (6)	0.9 (0.6, 1.3)	36 (6)	0.9 (0.6, 1.2)	0.55
Missing	1,134	27		42		45		
Trend (47.3)[h]			1.1 (1.0, 1.3)		1.0 (0.8, 1.1)		0.9 (0.8, 1.1)	0.15
Glyphosate								
0 (0.0)	4,371 (23)	94 (20)	Reference	113 (21)	Reference	143 (21)	Reference	
1-20 (8.8)	6,219 (32)	135 (29)	1.1 (0.8, 1.4)	203 (38)	1.3 (1.0, 1.6)	219 (32)	1.0 (0.8, 1.3)	
21-56 (38.8)	4,497 (23)	116 (25)	1.2 (0.9, 1.6)	107 (20)	0.9 (0.6, 1.1)	150 (22)	1.1 (0.8, 1.4)	
> 56 (116.0)	4,183 (22)	124 (26)	1.4 (1.0, 1.8)	112 (21)	1.0 (0.8, 1.3)	163 (24)	1.2 (0.9, 1.5)	0.04
Missing	236	5		5		13		
Trend (99.8)[h]			1.3 (1.1, 1.6)		0.9 (0.7, 1.1)		1.1 (1.0, 1.4)	0.02
Imazethapyr								
0 (0.0)	10,075 (55)	240 (54)	Reference	288 (58)	Reference	347 (54)	Reference	
1-9 (8.8)	3,733 (20)	91 (20)	1.1 (0.8, 1.5)	97 (20)	1.0 (0.7, 1.4)	129 (20)	1.2 (0.9, 1.6)	
10-25 (24.5)	2,639 (14)	61 (14)	1.0 (0.7, 1.3)	72 (14)	1.0 (0.7, 1.3)	105 (16)	1.2 (0.9, 1.5)	
> 25 (56.0)	1,975 (11)	53 (12)	1.1 (0.8, 1.5)	40 (8)	0.7 (0.5, 1.0)	66 (10)	1.0 (0.7, 1.3)	0.57
Missing	1,084	29		43		41		
Trend (30.0)[h]			1.0 (0.9, 1.2)		0.9 (0.7, 1.0)		1.0 (0.9, 1.1)	0.28

Cumulative lifetime days personally mixed or applied[a,b]	Non-cases [*n* (%)]	PRE-E[c] Cases [*n* (%)]	PRE-E[c] IP-weighted OR[d] (95% CI)	PRE-B[c] Cases [*n* (%)]	PRE-B[c] IP-weighted OR[d] (95% CI)	POST[c] Cases [*n* (%)]	POST[c] IP-weighted OR[d] (95% CI)	*p* for difference among ORs[e]
Metolachlor								
0 (0.0)	9,560 (52)	224 (50)	Reference	281 (56)	Reference	341 (52)	Reference	
1-25 (8.8)	4,309 (23)	101 (22)	1.1 (0.8, 1.4)	116 (23)	0.9 (0.7, 1.2)	139 (21)	1.0 (0.8, 1.2)	
26-56 (56.0)	1,898 (10)	47 (10)	1.0 (0.8, 1.4)	50 (10)	0.9 (0.7, 1.2)	78 (12)	1.1 (0.9, 1.5)	
> 56 (116.0)	2,732 (15)	79 (18)	1.2 (0.9, 1.6)	55 (11)	0.7 (0.5, 1.0)	92 (14)	1.0 (0.8, 1.2)	0.26
Missing	977	23		38		38		
Trend (88.5)[h]			1.1 (0.9, 1.4)		0.8 (0.6, 1.0)		1.0 (0.8, 1.2)	0.03
Paraquat[f]								
0 (0.0)	9,054 (84)	245 (82)	Reference	253 (85)	Reference	309 (86)	Reference	
1-9 (8.8)	911 (9)	30 (10)	1.6 (1.0, 2.4)	32 (11)	1.2 (0.7, 1.8)	25 (7)	0.7 (0.4, 1.2)	
10-25 (24.5)	309 (3)	11 (4)	1.2 (0.6, 2.4)	3 (1)	[i]	12 (3)	0.9 (0.5, 1.6)	
> 25 (108.5)	442 (4)	11 (4)	0.9 (0.5, 1.8)	10 (3)	0.7 (0.3, 1.3)	15 (4)	1.1 (0.6, 1.9)	0.13
Missing	274	9		17		10		
Trend (42.0)[h]			1.0 (0.8, 1.3)		0.8 (0.6, 1.1)		1.0 (0.8, 1.3)	0.56
Pendimethalin[f]								
0 (0.0)	6,752 (63)	182 (61)	Reference	192 (64)	Reference	249 (68)	Reference	
1-9 (8.8)	1,804 (17)	48 (16)	1.0 (0.7, 1.4)	63 (21)	1.2 (0.9, 1.6)	55 (15)	0.7 (0.6, 1.0)	
10-25 (24.5)	1,000 (9)	28 (9)	1.0 (0.6, 1.5)	18 (6)	0.5 (0.3, 0.9)	30 (8)	0.8 (0.5, 1.2)	
> 25 (56.0)	1,138 (11)	38 (13)	1.2 (0.8, 1.8)	26 (9)	0.8 (0.5, 1.2)	30 (8)	0.7 (0.5, 1.1)	0.06
Missing	296	10		16		7		
Trend (42.0)[h]			1.1 (0.9, 1.5)		0.8 (0.5, 1.1)		0.8 (0.6, 1.0)	0.09
Petroleum oil[f]								
0 (0.0)	8,295 (78)	223 (76)	Reference	218 (74)	Reference	272 (75)	Reference	
1-20 (8.8)	1,002 (9)	22 (8)	0.9 (0.6, 1.5)	34 (11)	1.2 (0.8, 1.7)	34 (9)	1.1 (0.7, 1.6)	
21-56 (38.8)	653 (6)	16 (5)	0.8 (0.5, 1.5)	18 (6)	1.0 (0.6, 1.6)	31 (9)	1.4 (1.0, 2.1)	
> 56 (224.8)	680 (6)	32 (11)	1.6 (1.1, 2.4)	26 (9)	1.4 (0.9, 2.1)	25 (7)	1.1 (0.7, 1.7)	0.50
Missing	360	13		19		9		
Trend (99.8)[h]			1.2 (1.0, 1.5)		1.1 (0.9, 1.4)		1.1 (0.9, 1.3)	0.51

Cumulative lifetime days personally mixed or applied[a,b]	Non-cases [*n* (%)]	PRE-E[c] Cases [*n* (%)]	PRE-E[c] IP-weighted OR[d] (95% CI)	PRE-B[c] Cases [*n* (%)]	PRE-B[c] IP-weighted OR[d] (95% CI)	POST[c] Cases [*n* (%)]	POST[c] IP-weighted OR[d] (95% CI)	*p* for difference among ORs[e]
Trifluralin								
0 (0.0)	8,343 (45)	183 (41)	Reference	218 (43)	Reference	285 (45)	Reference	
1-25 (20.0)	3,512 (19)	84 (19)	1.2 (0.9, 1.6)	109 (21)	1.2 (0.9, 1.5)	111 (17)	1.1 (0.8, 1.4)	
26-109 (56.0)	3,953 (21)	94 (21)	1.1 (0.9, 1.4)	111 (22)	1.1 (0.9, 1.4)	147 (23)	1.1 (0.9, 1.3)	
> 109 (224.8)	2,600 (14)	86 (19)	1.5 (1.1, 1.9)	73 (14)	1.1 (0.8, 1.4)	94 (15)	1.1 (0.8, 1.4)	0.61
Missing	1,098	27		29		51		
Trend (91.5)[h]			1.2 (1.0, 1.3)		1.0 (0.9, 1.1)		1.0 (0.9, 1.1)	0.19
Phenoxy herbicides								
2,4-D								
0 (0.0)	3,976 (21)	91 (20)	Reference	95 (18)	Reference	151 (23)	Reference	
1-39 (20.0)	5,950 (31)	100 (22)	0.7 (0.5, 1.0)	174 (33)	1.2 (0.9, 1.6)	210 (31)	1.0 (0.8, 1.2)	
40-116 (87.5)	4,629 (24)	135 (29)	1.3 (1.0, 1.7)	133 (25)	1.3 (1.0, 1.7)	161 (24)	0.9 (0.7, 1.2)	
> 116 (245.0)	4,575 (24)	137 (30)	1.3 (1.0, 1.7)	131 (25)	1.3 (1.0, 1.6)	147 (22)	0.9 (0.7, 1.1)	< 0.01
Missing	376	11		7		19		
Trend (154.0)[h]			1.3 (1.1, 1.5)		1.1 (0.9, 1.2)		0.9 (0.8, 1.1)	< 0.01
2,4,5-T[f]								
0 (0.0)	8,541 (80)	204 (70)	Reference	223 (74)	Reference	290 (81)	Reference	
1-9 (8.8)	1,240 (12)	52 (18)	1.9 (1.3, 2.7)	42 (14)	1.4 (0.9, 2.1)	44 (12)	1.1 (0.7, 1.5)	
10-25 (22.3)	382 (4)	21 (7)	2.2 (1.4, 3.6)	15 (5)	1.5 (0.9, 2.6)	11 (3)	0.8 (0.4, 1.5)	
> 25 (62.5)	518 (5)	16 (5)	1.3 (0.8, 2.3)	20 (7)	1.5 (0.9, 2.4)	14 (4)	0.7 (0.4, 1.3)	0.03
Missing	309	13		15		12		
Trend (30.0)[h]			1.3 (1.1, 1.6)		1.3 (1.0, 1.6)		0.9 (0.7, 1.1)	0.03
2,4,5-TP[f, g]								
0 (0.0)	10,114 (95)	268 (91)	Reference	281 (94)	Reference	338 (94)	Reference	
1-20 (8.8)	319 (3)	13 (4)	1.9 (1.0, 3.5)	12 (4)	1.5 (0.8, 3.1)	17 (5)	1.7 (1.0, 3.0)	
> 20 (56.0)	223 (2)	12 (4)	2.1 (1.1, 3.9)	7 (2)	1.1 (0.5, 2.5)	5 (1)	0.6 (0.2, 1.5)	0.23
Missing	334	13		15		11		
Trend (47.4)[h]			2.0 (1.2, 3.2)		1.2 (0.6, 2.2)		0.8 (0.4, 1.5)	0.05

Cumulative lifetime days personally mixed or applied[a,b]	Non-cases [*n* (%)]	PRE-E[c] Cases [*n* (%)]	PRE-E[c] IP-weighted OR[d] (95% CI)	PRE-B[c] Cases [*n* (%)]	PRE-B[c] IP-weighted OR[d] (95% CI)	POST[c] Cases [*n* (%)]	POST[c] IP-weighted OR[d] (95% CI)	*p* for difference among ORs[e]
Triazine herbicides								
Atrazine								
0 (0.0)	4,854 (25)	100 (21)	Reference	122 (23)	Reference	173 (26)	Reference	
1-25 (8.8)	4,748 (25)	105 (22)	1.0 (0.8, 1.4)	120 (23)	1.0 (0.7, 1.2)	179 (26)	1.1 (0.9, 1.4)	
26-109 (56.0)	5,353 (28)	131 (28)	1.2 (0.9, 1.6)	175 (33)	1.3 (1.0, 1.7)	159 (23)	0.8 (0.7, 1.1)	
> 109 (224.8)	4,285 (22)	133 (28)	1.5 (1.2, 2.0)	116 (22)	1.0 (0.8, 1.4)	166 (25)	1.1 (0.9, 1.4)	< 0.01
Missing	266	5		7		11		
Trend (158.5)[h]			1.3 (1.1, 1.5)		1.0 (0.9, 1.2)		1.1 (0.9, 1.2)	0.11
Cyanazine								
0 (0.0)	10,284 (55)	222 (49)	Reference	259 (50)	Reference	350 (54)	Reference	
1-20 (8.8)	3,375 (18)	77 (17)	1.0 (0.7, 1.4)	92 (18)	1.0 (0.7, 1.3)	128 (20)	1.2 (1.0, 1.6)	
21-56 (38.8)	2,611 (14)	79 (17)	1.4 (1.1, 1.9)	89 (17)	1.3 (1.0, 1.7)	90 (14)	1.0 (0.8, 1.3)	
> 56 (116.0)	2,276 (12)	74 (16)	1.5 (1.2, 2.0)	75 (15)	1.3 (1.0, 1.8)	82 (13)	1.1 (0.8, 1.3)	0.05
Missing	960	22		25		38		
Trend (99.8)[h]			1.4 (1.2, 1.8)		1.3 (1.0, 1.6)		1.0 (0.8, 1.3)	0.10
Metribuzin[f]								
0 (0.0)	6,347 (59)	169 (57)	Reference	160 (54)	Reference	226 (62)	Reference	
1-9 (8.8)	2,099 (20)	65 (22)	1.3 (0.9, 1.9)	67 (23)	1.0 (0.7, 1.4)	57 (16)	0.8 (0.5, 1.1)	
10-25 (24.5)	1,110 (10)	27 (9)	0.9 (0.6, 1.3)	35 (12)	1.3 (0.9, 1.8)	44 (12)	1.1 (0.8, 1.5)	
> 25 (56.0)	1,121 (11)	35 (12)	1.2 (0.8, 1.8)	34 (11)	1.2 (0.8, 1.8)	35 (10)	0.9 (0.6, 1.3)	0.23
Missing	313	10		19		9		
Trend (30.0)[h]			1.1 (0.9, 1.3)		1.1 (0.9, 1.3)		0.9. (0.8, 1.1)	0.47
Insecticides								
Carbamates[j]								
Aldicarb[f, g]								
0 (0.0)	9,974 (93)	272 (92)	Reference	280 (94)	Reference	332 (91)	Reference	
1-25 (8.8)	425 (4)	17 (6)	1.8 (0.8, 3.7)	15 (5)	1.9 (0.9, 4.1)	22 (6)	2.2 (1.2, 4.1)	
> 25 (108.5)	298 (3)	6 (2)	1.1 (0.5, 2.7)	4 (1)	[i]	9 (2)	1.1 (0.5, 2.2)	0.66
Missing	293	11		16		8		
Trend (99.8)[h]			1.1 (0.5, 2.4)		0.5 (0.2, 1.2)		1.1 (0.6, 2.0)	0.32

Cumulative lifetime days personally mixed or applied[a,b]	Non-cases [*n* (%)]	PRE-E[c] Cases [*n* (%)]	PRE-E[c] IP-weighted OR[d] (95% CI)	PRE-B[c] Cases [*n* (%)]	PRE-B[c] IP-weighted OR[d] (95% CI)	POST[c] Cases [*n* (%)]	POST[c] IP-weighted OR[d] (95% CI)	*p* for difference among ORs[e]
Carbaryl[f]								
0 (0.0)	6,033 (57)	160 (54)	Reference	154 (52)	Reference	206 (57)	Reference	
1-9 (8.8)	1,892 (18)	58 (20)	1.2 (0.9, 1.7)	68 (23)	1.4 (1.0, 1.9)	62 (17)	0.9 (0.7, 1.3)	
10-56 (24.5)	1,521 (14)	32 (11)	1.0 (0.7, 1.6)	32 (11)	0.8 (0.5, 1.3)	50 (14)	0.9 (0.7, 1.3)	
> 56 (175.0)	1,190 (11)	46 (16)	1.7 (1.2, 2.4)	41 (14)	1.4 (1.0, 2.0)	44 (12)	1.1 (0.7, 1.5)	0.36
Missing	354	10		20		9		
Trend (99.8)[h]			1.3 (1.1, 1.6)		1.2 (1.0, 1.5)		1.1 (0.9, 1.3)	0.29
Carbofuran								
0 (0.0)	12,998 (71)	296 (67)	Reference	331 (65)	Reference	468 (73)	Reference	
1-9 (8.8)	2,245 (12)	53 (12)	1.0 (0.8, 1.4)	77 (15)	1.3 (1.0, 1.7)	69 (11)	0.9 (0.7, 1.2)	
10-51 (24.5)	1,970 (11)	57 (13)	1.3 (1.0, 1.7)	63 (12)	1.3 (1.0, 1.7)	71 (11)	1.0 (0.8, 1.3)	
> 51 (116.0)	1,224 (7)	39 (9)	1.3 (0.9, 1.9)	35 (7)	1.1 (0.8, 1.6)	36 (6)	0.9 (0.6, 1.2)	0.23
Missing	1,069	29		34		44		
Trend (47.3)[h]			1.1 (1.0, 1.3)		1.0 (0.9, 1.2)		0.9 (0.8, 1.1)	0.14
Organochlorine insecticides								
Aldrin[f]								
0 (0.0)	8,722 (82)	214 (73)	Reference	218 (74)	Reference	293 (81)	Reference	
1-9 (8.8)	904 (8)	31 (11)	1.7 (1.0, 2.8)	31 (10)	1.5 (0.9, 2.5)	44 (12)	1.6 (1.1, 2.4)	
10-25 (24.5)	576 (5)	35 (12)	2.5 (1.7, 3.7)	27 (9)	1.9 (1.3, 3.0)	15 (4)	0.7 (0.4, 1.2)	
> 25 (56.0)	464 (4)	15 (5)	1.2 (0.7, 2.1)	20 (7)	1.7 (1.1, 2.7)	9 (2)	0.6 (0.3, 1.1)	< 0.01
Missing	324	11		19		10		
Trend (15.8)[h]			1.2 (1.1, 1.3)		1.2 (1.1, 1.4)		0.9 (0.8, 1.0)	< 0.01
Chlordane[f]								
0 (0.0)	8,480 (80)	205 (70)	Reference	224 (76)	Reference	285 (79)	Reference	
1-9 (8.8)	1,464 (14)	58 (20)	2.0 (1.4, 2.8)	45 (15)	1.1 (0.7, 1.6)	53 (15)	1.3 (0.9, 1.8)	
10-20 (20.0)	189 (2)	4 (1)	[i]	6 (2)	1.5 (0.6, 3.4)	8 (2)	1.1 (0.5, 2.4)	
> 20 (50.8)	522 (5)	27 (9)	2.0 (1.3, 3.0)	21 (7)	1.7 (1.0, 2.7)	15 (4)	0.7 (0.4, 1.3)	0.01
Missing	335	12		19		10		
Trend (22.0)[h]			1.4 (1.2, 1.6)		1.3 (1.0, 1.6)		0.9 (0.7, 1.1)	0.01

**Cumulative lifetime days personally mixed or applied**[a,b]	**Non-cases [*n* (%)]**	**PRE-E**[c] **Cases [*n* (%)]**	**PRE-E**[c] **IP-weighted OR**[d] **(95% CI)**	**PRE-B**[c] **Cases [*n* (%)]**	**PRE-B**[c] **IP-weighted OR**[d] **(95% CI)**	**POST**[c] **Cases [*n* (%)]**	**POST**[c] **IP-weighted OR**[d] **(95% CI)**	***p* for difference among ORs**[e]
DDT[f]								
0 (Median = 0.0)	8,159 (77)	205 (70)	Reference	219 (74)	Reference	284 (80)	Reference	
1-9 (8.8)	1,147 (11)	34 (12)	1.1 (0.7, 1.7)	29 (10)	1.3 (0.8, 2.3)	34 (10)	1.1 (0.7, 1.9)	
10-39 (24.5)	571 (5)	21 (7)	1.6 (1.0, 2.5)	17 (6)	1.1 (0.7, 1.8)	17 (5)	0.9 (0.6, 1.6)	
> 39 (108.5)	761 (7)	31 (11)	1.7 (1.1, 2.5)	30 (10)	1.7 (1.1, 2.5)	23 (6)	0.8 (0.5, 1.3)	0.19
Missing	352	15		20		13		
Trend (47.3)[h]			1.3 (1.1, 1.5)		1.2 (1.0, 1.5)		0.9 (0.8, 1.1)	0.02
Dieldrin[f,g]								
0 (0.0)	10,219 (96)	276 (95)	Reference	284 (96)	Reference	350 (96)	Reference	
1-9 (8.8)	279 (3)	9 (3)	1.0 (0.5, 2.0)	10 (3)	1.2 (0.5, 3.0)	9 (2)	1.3 (0.5, 3.1)	
> 9 (24.5)	152 (1)	7 (2)	1.5 (0.7, 3.3)	3 (1)	[i]	5 (1)	1.3 (0.5, 3.4)	0.70
Missing	340	14		18		7		
Trend (15.8)[h]			1.4 (0.9, 2.1)		0.9 (0.5, 1.5)		1.1 (0.6, 2.0)	0.50
Heptachlor[f]								
0 (0.0)	9,283 (87)	226 (76)	Reference	253 (84)	Reference	314 (86)	Reference	
1-9 (8.8)	731 (7)	28 (9)	1.6 (0.9, 2.6)	27 (9)	1.4 (0.8, 2.5)	25 (7)	1.3 (0.7, 2.1)	
10-25 (24.5)	387 (4)	23 (8)	2.5 (1.6, 3.9)	14 (5)	1.3 (0.8, 2.3)	14 (4)	1.1 (0.6, 1.8)	
> 25 (56.0)	291 (3)	21 (7)	3.1 (1.9, 5.0)	7 (2)	0.8 (0.4, 1.8)	11 (3)	1.2 (0.6, 2.2)	0.01
Missing	298	8		14		7		
Trend (15.8)[h]			1.4 (1.3, 1.6)		1.0 (0.9, 1.2)		1.1 (0.9, 1.2)	< 0.01
Lindane[f]								
0 (0.0)	9,029 (85)	232 (79)	Reference	223 (76)	Reference	287 (80)	Reference	
1-9 (8.8)	740 (7)	19 (6)	1.0 (0.6, 1.7)	40 (14)	2.0 (1.4, 3.0)	34 (9)	1.4 (0.9, 2.2)	
10-25 (20.0)	340 (3)	20 (7)	2.1 (1.3, 3.4)	7 (2)	0.8 (0.3, 1.7)	16 (4)	1.6 (0.9, 2.7)	
> 25 (103.3)	527 (5)	24 (8)	1.7 (1.1, 2.6)	25 (8)	2.0 (1.3, 3.1)	23 (6)	1.4 (0.9, 2.1)	0.07
Missing	354	11		20		11		
Trend (47.3)[h]			1.3 (1.1, 1.6)		1.4 (1.1, 1.7)		1.2 (1.0, 1.4)	0.54
Toxaphene[f]								
0 (0.0)	9,422 (88)	247 (83)	Reference	255 (85)	Reference	318 (87)	Reference	
1-9 (8.8)	655 (6)	26 (9)	1.4 (0.9, 2.2)	22 (7)	1.2 (0.8, 2.0)	21 (6)	0.9 (0.6, 1.6)	

Cumulative lifetime days personally mixed or applied[a,b]	Non-cases [*n* (%)]	PRE-E[c] Cases [*n* (%)]	PRE-E[c] IP-weighted OR[d] (95% CI)	PRE-B[c] Cases [*n* (%)]	PRE-B[c] IP-weighted OR[d] (95% CI)	POST[c] Cases [*n* (%)]	POST[c] IP-weighted OR[d] (95% CI)	*p* for difference among ORs[e]
10-25 (24.5)	283 (3)	12 (4)	1.4 (0.7, 2.5)	11 (4)	1.5 (0.8, 2.9)	15 (4)	1.5 (0.9, 2.7)	
> 25 (108.5)	331 (3)	14 (5)	1.5 (0.9, 2.7)	12 (4)	1.5 (0.8, 2.8)	11 (3)	0.9 (0.5, 1.7)	0.76
Missing	299	7		15		6		
Trend (42.0)[h]			1.2 (1.0, 1.5)		1.2 (0.9, 1.5)		1.0 (0.8, 1.2)	0.32
Organophosphate insecticides								
Chlorpyrifos								
0 (0.0)	10,913 (57)	252 (54)	Reference	267 (50)	Reference	379 (56)	Reference	
1-20 (8.8)	3,707 (19)	107 (23)	1.3 (1.0, 1.7)	118 (22)	1.4 (1.1, 1.7)	132 (20)	1.0 (0.8, 1.3)	
21-56 (38.8)	2,850 (15)	62 (13)	0.9 (0.7, 1.2)	94 (18)	1.3 (1.0, 1.7)	107 (16)	1.1 (0.9, 1.4)	
> 56 (116.0)	1,785 (9)	47 (10)	1.1 (0.8, 1.6)	54 (10)	1.1 (0.8, 1.5)	57 (8)	0.9 (0.7, 1.2)	0.24
Missing	251	6		7		13		
Trend (47.3)[h]			1.0 (0.9, 1.2)		1.1 (0.9, 1.2)		1.0 (0.9, 1.1)	0.67
Coumaphos								
0 (0.0)	16,483 (90)	387 (88)	Reference	437 (88)	Reference	576 (92)	Reference	
1-9 (8.8)	777 (4)	16 (4)	0.8 (0.5, 1.4)	26 (5)	1.2 (0.8, 1.8)	27 (4)	0.9 (0.6, 1.3)	
10-39 (24.5)	490 (3)	18 (4)	1.5 (1.0, 2.5)	18 (4)	1.4 (0.9, 2.3)	16 (3)	0.9 (0.5, 1.5)	
> 39 (116.0)	480 (3)	18 (4)	1.5 (0.9, 2.4)	16 (3)	1.2 (0.7, 2.1)	8 (1)	0.5 (0.2, 1.0)	0.10
Missing	1,276	35		43		61		
Trend (47.3)[h]			1.2 (1.0, 1.4)		1.1 (0.9, 1.4)		0.8 (0.6, 1.0)	0.02
Diazinon[f]								
0 (0.0)	8,386 (79)	229 (78)	Reference	209 (71)	Reference	271 (75)	Reference	
1-9 (8.8)	1,107 (10)	34 (12)	1.2 (0.8, 1.8)	44 (15)	1.7 (1.2, 2.5)	43 (12)	1.2 (0.8, 1.7)	
10-25 (24.5)	536 (5)	13 (4)	0.7 (0.4, 1.3)	14 (5)	1.0 (0.5, 1.8)	18 (5)	1.2 (0.7, 1.9)	
> 25 (103.3)	600 (6)	19 (6)	1.2 (0.7, 1.9)	27 (9)	1.9 (1.2, 2.9)	29 (8)	1.5 (1.0, 2.2)	0.42
Missing	361	11		21		10		
Trend (42.0)[h]			1.1 (0.9, 1.3)		1.3 (1.1, 1.5)		1.2 (1.0, 1.4)	0.39
Dichlorvos								
0 (0.0)	16,229 (88)	386 (86)	Reference	410 (82)	Reference	549 (85)	Reference	
1-20 (8.8)	847 (5)	22 (5)	1.3 (0.8, 2.1)	30 (6)	1.3 (0.8, 1.9)	36 (6)	1.3 (0.9, 1.9)	

Cumulative lifetime days personally mixed or applied[a,b]	Non-cases [*n* (%)]	PRE-E[c] Cases [*n* (%)]	PRE-E[c] IP-weighted OR[d] (95% CI)	PRE-B[c] Cases [*n* (%)]	PRE-B[c] IP-weighted OR[d] (95% CI)	POST[c] Cases [*n* (%)]	POST[c] IP-weighted OR[d] (95% CI)	*p* for difference among ORs[e]
21-116 (56.0)	759 (4)	24 (5)	1.3 (0.9, 2.0)	37 (7)	2.0 (1.4, 2.9)	32 (5)	1.3 (0.9, 1.8)	
> 116 (457.3)	634 (3)	15 (3)	0.9 (0.6, 1.6)	24 (5)	1.5 (1.0, 2.2)	28 (4)	1.3 (0.9, 1.9)	0.45
Missing	1,037	27		39		43		
Trend (216.0)[h]			1.0 (0.8, 1.2)		1.2 (1.0, 1.5)		1.1 (0.9, 1.4)	0.35
Fonofos								
0 (0.0)	14,176 (77)	319 (71)	Reference	369 (72)	Reference	498 (78)	Reference	
1-20 (8.8)	1,791 (10)	50 (11)	1.2 (0.9, 1.7)	57 (11)	1.2 (0.9, 1.6)	54 (8)	0.9 (0.6, 1.2)	
21-51 (24.5)	1,206 (7)	45 (10)	1.6 (1.1, 2.2)	39 (8)	1.2 (0.9, 1.7)	46 (7)	1.0 (0.8, 1.4)	
> 51 (116.0)	1,321 (7)	34 (8)	1.1 (0.8, 1.6)	46 (9)	1.4 (1.0, 1.9)	43 (7)	0.9 (0.7, 1.3)	0.24
Missing	1,012	26		29		47		
Trend (47.3)[h]			1.1 (0.9, 1.2)		1.1 (1.0, 1.3)		1.0 (0.8, 1.1)	0.20
Malathion[f]								
0 (0.0)	3,559 (34)	77 (26)	Reference	74 (25)	Reference	109 (31)	Reference	
1-9 (8.8)	2,871 (27)	74 (25)	1.1 (0.8, 1.6)	71 (24)	1.2 (0.8, 1.7)	102 (29)	1.0 (0.8, 1.4)	
10-39 (24.5)	2,253 (21)	65 (22)	1.3 (0.9, 1.8)	79 (27)	1.6 (1.2, 2.3)	73 (21)	1.0 (0.7, 1.4)	
> 39 (108.5)	1,939 (18)	77 (26)	1.8 (1.3, 2.5)	72 (24)	1.9 (1.4, 2.7)	71 (20)	1.2 (0.8, 1.6)	0.21
Missing	368	13		19		16		
Trend (47.3)[h]			1.3 (1.1, 1.4)		1.3 (1.1, 1.4)		1.1 (0.9, 1.2)	0.07
Parathion[f]								
0 (0.0)	9,784 (92)	257 (88)	Reference	262 (89)	Reference	336 (93)	Reference	
1-9 (8.8)	362 (3)	16 (6)	1.5 (0.9, 2.8)	12 (4)	0.8 (0.4, 1.7)	15 (4)	1.3 (0.7, 2.3)	
10-51 (24.5)	229 (2)	8 (3)	1.5 (0.6, 3.4)	8 (3)	1.0 (0.5, 2.1)	6 (2)	0.6 (0.3, 1.5)	
> 51 (116.0)	236 (2)	10 (3)	1.4 (0.7, 2.8)	13 (4)	2.3 (1.3, 4.2)	5 (1)	0.8 (0.3, 1.9)	0.24
Missing	379	15		20		9		
Trend (53.8)[h]			1.2 (0.9, 1.6)		1.5 (1.1, 1.9)		0.9 (0.6, 1.4)	0.13
Phorate[f]								
0 (0.0)	7,258 (68)	167 (57)	Reference	189 (64)	Reference	259 (71)	Reference	
1-9 (8.8)	1,452 (14)	42 (14)	1.4 (0.9, 2.0)	49 (17)	1.1 (0.8, 1.6)	41 (11)	0.7 (0.5, 1.0)	
10-25 (24.5)	961 (9)	39 (13)	1.6 (1.1, 2.3)	33 (11)	1.3 (0.9, 1.8)	27 (7)	0.7 (0.5, 1.1)	
> 25 (62.5)	990 (9)	44 (15)	1.8 (1.3, 2.6)	25 (8)	0.9 (0.6, 1.4)	36 (10)	1.0 (0.7, 1.4)	< 0.01

Cumulative lifetime days personally mixed or applied[a,b]	Non-cases [*n* (%)]	PRE-E[c] Cases [*n* (%)]	PRE-E[c] IP-weighted OR[d] (95% CI)	PRE-B[c] Cases [*n* (%)]	PRE-B[c] IP-weighted OR[d] (95% CI)	POST[c] Cases [*n* (%)]	POST[c] IP-weighted OR[d] (95% CI)	*p* for difference among ORs[e]
Missing	329	14		19		8		
Trend (47.3)[h]			1.6 (1.2, 2.0)		1.0 (0.7, 1.4)		0.9 (0.7, 1.2)	0.01
Terbufos								
0 (0.0)	10,867 (59)	227 (51)	Reference	274 (54)	Reference	385 (60)	Reference	
1-20 (8.8)	2,755 (15)	73 (16)	1.3 (1.0, 1.7)	89 (18)	1.2 (0.9, 1.6)	92 (14)	1.0 (0.8, 1.2)	
21-56 (38.8)	2,639 (14)	75 (17)	1.4 (1.0, 1.8)	77 (15)	1.1 (0.9, 1.5)	97 (15)	1.0 (0.8, 1.3)	
> 56 (116.0)	2,208 (12)	71 (16)	1.5 (1.1, 1.9)	65 (13)	1.2 (0.9, 1.6)	69 (11)	0.9 (0.7, 1.2)	0.19
Missing	1,037	28		35		45		
Trend (88.5)[h]			1.3 (1.1, 1.6)		1.1 (0.9, 1.4)		0.9 (0.8, 1.2)	0.07
Pyrethroid insecticides								
Permethrin (for animals)								
0 (0.0)	15,726 (85)	372 (83)	Reference	420 (83)	Reference	546 (84)	Reference	
1-9 (8.8)	1,195 (6)	25 (6)	0.9 (0.6, 1.4)	37 (7)	1.0 (0.7, 1.5)	37 (6)	1.0 (0.7, 1.5)	
10-51 (24.5)	868 (5)	18 (4)	0.8 (0.5, 1.4)	29 (6)	1.3 (0.9, 1.9)	30 (5)	1.0 (0.7, 1.4)	
> 51 (116.0)	737 (4)	33 (7)	1.8 (1.2, 2.6)	21 (4)	1.0 (0.7, 1.6)	34 (5)	1.4 (1.0, 1.9)	0.43
Missing	980	26		33		41		
Trend (47.3)[h]			1.3 (1.1, 1.5)		1.0 (0.9, 1.2)		1.1 (1.0, 1.3)	0.26
Permethrin (for crops)								
0 (0.0)	15,902 (87)	377 (85)	Reference	415 (84)	Reference	560 (88)	Reference	
1-9 (8.8)	1,294 (7)	37 (8)	1.3 (0.9, 1.8)	48 (10)	1.5 (1.1, 2.1)	46 (7)	1.0 (0.7, 1.4)	
10-30 (24.5)	481 (3)	14 (3)	1.3 (0.8, 2.4)	15 (3)	1.1 (0.7, 2.0)	5 (1)	0.3 (0.1, 0.7)	
> 30 (108.5)	685 (4)	15 (3)	0.9 (0.5, 1.6)	15 (3)	0.9 (0.5, 1.5)	27 (4)	1.2 (0.8, 1.8)	0.04
Missing	1,144	31		47		50		
Trend (47.3)[h]			1.0 (0.8, 1.2)		1.0 (0.8, 1.2)		1.0 (0.9, 1.3)	0.88

Abbreviations: 2,4-D, (2,4-dichlorophenoxy)acetic acid; 2,4,5-T, (2,4,5-trichlorophenoxy)acetic acid; 2,4,5-TP, (*RS*)-2-(2,4,5-trichlorophenoxy)propionic acid; CI, confidence interval; DDT, 1,1,1-trichloro-2,2-bis(4-chlorophenyl)ethane; EPTC, *S*-ethyl dipropyl(thiocarbamate); IQR, interquartile range; IP, inverse probability; OR, odds ratio; POST, post-enrollment; PRE-B, pre-enrollment both; PRE-E, pre-enrollment enrollment only.

[a]Category boundaries set at tertiles of cumulative lifetime days of use for each pesticide among all male private pesticide applicators who used it. [b]Fewer than five PRE-E, PRE-B, or POST cases used trichlorfon or ziram at every exposure level. [c]See footnote a at the bottom of Table S1 for a description of the three case groups. [d]Weights adjusted for age at enrollment (modeled with a restricted, quadratic spline with knots at ages 40, 48, and 57 years based on percentiles of the age distribution in cases), ever diagnosed with diabetes, education level, state of residence, not missing covariate data (conditional on age, state, the exposure, and pairwise interaction terms between each covariate and the exposure), and not dropping out of the AHS cohort (conditional on age, diabetes, education, state, the exposure, and pairwise interaction terms between each covariate and the exposure). 95% CIs calculated with robust variance estimates. [e]Differences among case-group-specific ORs tested via Wald χ^2 tests. [f]Data available only for 11,982 applicators who completed the farmer questionnaire. Weights additionally adjusted for completing the farmer questionnaire (conditional on age, diabetes, education, and state). [g]Category boundaries set at the median of cumulative lifetime days of use for each pesticide among all male private pesticide applicators who used it. [h]Used within-category medians and scaled the OR to an IQR-unit (days) increase in cumulative lifetime days of use for each pesticide among all male private pesticide applicators who used it. [i]OR (95% CI) not shown because fewer than five PRE-E or PRE-B cases used aldicarb, captan, carbon tetrachloride/carbon disulfide (80/20 mix), chlordane, dieldrin, ethylene dibromide, maneb/mancozeb, or paraquat at this exposure level. [j]Benomyl is also included in carbamates.

Table S6. Pesticide use and self-reported depression among male private pesticide applicators in the Agricultural Health Study without weighting for potential biases from missing covariate data, missing farmer questionnaire, or drop out.

Variable	PRE-E[a] Adjusted OR[b] (95% CI)	PRE-B[a] Adjusted OR[b] (95% CI)	POST[a] Adjusted OR[b] (95% CI)	*p* for Difference among ORs[c]
Cumulative days personally mixed or applied pesticides[d]				
≤ 56 (Median = 24.5)	Reference	Reference	Reference	
57-225 (116.0)	1.3 (1.0, 1.7)	1.1 (0.9, 1.4)	0.9 (0.7, 1.1)	
226-457 (369.8)	1.4 (1.0, 1.8)	1.2 (0.9, 1.6)	1.1 (0.9, 1.4)	
> 457 (767.3)	1.5 (1.1, 2.0)	1.1 (0.8, 1.5)	1.0 (0.8, 1.2)	0.29
Missing				
Trend (IQR = 401.3)[e]	1.2 (1.0, 1.3)	1.0 (0.9, 1.2)	1.0 (0.9, 1.2)	0.26
Ever diagnosed with pesticide poisoning[f]				
No	Reference	Reference	Reference	
Yes	5.1 (3.4, 7.7)	2.7 (1.6, 4.5)	1.0 (0.5, 2.2)	< 0.01
Missing				
Ever experienced an incident of unusually high personal pesticide exposure[f]				
No	Reference	Reference	Reference	
Yes	2.3 (1.8, 3.0)	2.2 (1.7, 2.8)	1.1 (0.8, 1.4)	< 0.01
Missing				

Abbreviations: CI, confidence interval; IQR, interquartile range; OR, odds ratio; POST, post-enrollment; PRE-B, pre-enrollment both; PRE-E, pre-enrollment enrollment only.

[a]See footnote a at the bottom of Table S1 for a description of the three case groups. [b]Adjusted for age at enrollment (modeled with a cubic polynomial), ever diagnosed with diabetes, education level, and state of residence. [c]Differences among case-group-specific ORs tested via Wald χ^2 tests. [d]Category boundaries set at quartiles of cumulative days of pesticide use among all male private pesticide applicators. [e]Used within-category medians and scaled the OR to an IQR-unit (days) increase in cumulative days of pesticide use among all male private pesticide applicators. [f]Data available only for 11,982 applicators who completed the farmer questionnaire.

Table S7. Ever-use of pesticide classes and specific pesticides and self-reported depression among male private pesticide applicators in the Agricultural Health Study without weighting for potential biases from missing covariate data, missing farmer questionnaire, or drop out.

Ever personally mixed or applied	PRE-E[a] Adjusted OR[b,c] (95% CI)	PRE-B[a] Adjusted OR[b,c] (95% CI)	POST[a] Adjusted OR[b,c] (95% CI)	*p* for Difference among ORs[d]
Fumigants	1.3 (1.1, 1.7)	1.7 (1.4, 2.1)	1.2 (1.0, 1.5)	0.04
Aluminum phosphide	1.5 (1.0, 2.1)	1.5 (1.1, 2.1)	1.5 (1.1, 2.0)	1.00
Carbon tetrachloride/ carbon disulfide (80/20 mix)	1.5 (1.1, 2.0)	1.6 (1.2, 2.2)	1.2 (0.9, 1.7)	0.40
Ethylene dibromide	1.5 (1.0, 2.2)	1.4 (0.9, 2.1)	1.2 (0.8, 1.8)	0.83
Methyl bromide	1.2 (0.9, 1.6)	1.4 (1.0, 1.8)	1.0 (0.8, 1.3)	0.31
Fungicides	1.2 (1.0, 1.5)	1.4 (1.1, 1.7)	1.1 (0.9, 1.3)	0.24
Benomyl[e]	1.1 (0.8, 1.6)	1.0 (0.7, 1.4)	1.1 (0.8, 1.4)	0.82
Captan	1.1 (0.9, 1.5)	1.4 (1.1, 1.8)	1.1 (0.9, 1.4)	0.29
Chlorothalonil	1.0 (0.7, 1.5)	1.4 (1.0, 1.9)	1.1 (0.8, 1.5)	0.49
Maneb/mancozeb	1.2 (0.9, 1.7)	1.1 (0.8, 1.5)	1.0 (0.8, 1.3)	0.70
Metalaxyl	1.4 (1.1, 1.8)	1.2 (1.0, 1.5)	1.0 (0.8, 1.2)	0.09
Ziram	1.5 (0.8, 2.9)	0.7 (0.3, 1.6)	1.2 (0.7, 2.2)	0.32
Herbicides	1.7 (0.7, 4.2)	1.3 (0.6, 2.8)	1.4 (0.7, 2.6)	0.90
Alachlor	1.2 (1.0, 1.5)	1.2 (1.0, 1.4)	1.1 (1.0, 1.3)	0.81
Butylate	1.0 (0.8, 1.2)	1.1 (0.9, 1.3)	1.1 (0.9, 1.3)	0.72
Chlorimuron-ethyl	1.0 (0.8, 1.2)	1.1 (0.9, 1.3)	1.1 (0.9, 1.3)	0.63
Dicamba	0.9 (0.7, 1.1)	1.0 (0.8, 1.3)	1.1 (0.9, 1.3)	0.47
EPTC	1.2 (1.0, 1.5)	0.9 (0.7, 1.1)	1.2 (1.0, 1.4)	0.13
Glyphosate	1.2 (1.0, 1.5)	1.1 (0.9, 1.4)	1.1 (0.9, 1.3)	0.72
Imazethapyr	1.1 (0.9, 1.3)	0.9 (0.7, 1.0)	1.1 (0.9, 1.3)	0.21
Metolachlor	1.1 (0.9, 1.3)	0.8 (0.7, 1.0)	1.0 (0.8, 1.1)	0.14
Paraquat	1.2 (0.9, 1.5)	1.1 (0.9, 1.4)	1.0 (0.8, 1.2)	0.67
Pendimethalin	1.2 (1.0, 1.4)	0.9 (0.8, 1.1)	0.9 (0.8, 1.1)	0.10
Petroleum oil	1.3 (1.1, 1.6)	1.2 (1.0, 1.5)	1.0 (0.9, 1.2)	0.18
Trifluralin	1.2 (1.0, 1.4)	1.1 (0.9, 1.3)	1.1 (0.9, 1.3)	0.73
Phenoxy herbicides	1.1 (0.8, 1.4)	1.2 (0.9, 1.6)	0.9 (0.8, 1.1)	0.24
2,4-D	1.0 (0.8, 1.3)	1.1 (0.9, 1.4)	0.9 (0.8, 1.1)	0.57
2,4,5-T	1.4 (1.2, 1.8)	1.5 (1.2, 1.8)	1.1 (0.9, 1.4)	0.09
2,4,5-TP	1.5 (1.1, 1.9)	1.4 (1.1, 1.8)	1.1 (0.8, 1.4)	0.26
Triazine herbicides	1.1 (0.8, 1.4)	1.0 (0.8, 1.2)	1.0 (0.8, 1.3)	0.82
Atrazine	1.2 (0.9, 1.5)	1.0 (0.8, 1.3)	1.0 (0.8, 1.2)	0.54
Cyanazine	1.3 (1.0, 1.6)	1.1 (0.9, 1.4)	1.1 (0.9, 1.3)	0.63
Metribuzin	1.1 (0.9, 1.3)	1.0 (0.9, 1.3)	1.0 (0.9, 1.2)	0.91

Ever personally mixed or applied	PRE-E[a] Adjusted OR[b,c] (95% CI)	PRE-B[a] Adjusted OR[b,c] (95% CI)	POST[a] Adjusted OR[b,c] (95% CI)	*p* for Difference among ORs[d]
Insecticides	1.4 (0.8, 2.4)	0.9 (0.6, 1.4)	1.5 (1.0, 2.4)	0.20
Carbamates[e]	1.1 (0.9, 1.3)	1.2 (1.0, 1.5)	1.1 (0.9, 1.4)	0.68
Aldicarb	0.9 (0.7, 1.3)	1.1 (0.8, 1.5)	1.2 (0.9, 1.6)	0.45
Carbaryl	1.2 (1.0, 1.5)	1.3 (1.1, 1.6)	1.2 (1.0, 1.4)	0.75
Carbofuran	1.1 (0.9, 1.3)	1.2 (1.0, 1.4)	0.9 (0.8, 1.1)	0.16
Organochlorine insecticides	1.9 (1.5, 2.3)	1.2 (1.0, 1.5)	1.2 (1.0, 1.5)	0.01
Aldrin	1.4 (1.1, 1.7)	1.5 (1.2, 1.8)	1.2 (1.0, 1.5)	0.40
Chlordane	1.5 (1.3, 1.9)	1.2 (1.0, 1.5)	1.1 (0.9, 1.3)	0.05
DDT	1.3 (1.1, 1.7)	1.2 (0.9, 1.4)	0.9 (0.7, 1.1)	0.03
Dieldrin	1.4 (1.0, 1.9)	1.3 (1.0, 1.8)	1.1 (0.8, 1.5)	0.60
Heptachlor	1.6 (1.3, 2.0)	1.3 (1.0, 1.6)	1.0 (0.8, 1.2)	0.01
Lindane	1.6 (1.3, 1.9)	1.3 (1.0, 1.5)	1.1 (0.9, 1.4)	0.05
Toxaphene	1.3 (1.0, 1.6)	1.4 (1.1, 1.7)	1.1 (0.9, 1.4)	0.49
Organophosphate insecticides	1.5 (1.0, 2.1)	1.1 (0.8, 1.5)	1.3 (1.0, 1.8)	0.51
Chlorpyrifos	1.2 (1.0, 1.4)	1.3 (1.1, 1.6)	1.0 (0.9, 1.2)	0.07
Coumaphos	1.3 (1.0, 1.7)	1.3 (1.0, 1.6)	0.9 (0.7, 1.1)	0.08
Diazinon	1.3 (1.1, 1.6)	1.4 (1.1, 1.6)	1.1 (1.0, 1.4)	0.39
Dichlorvos	1.0 (0.8, 1.4)	1.5 (1.2, 1.9)	1.3 (1.1, 1.6)	0.10
Fonofos	1.3 (1.0, 1.6)	1.2 (0.9, 1.4)	1.0 (0.8, 1.2)	0.17
Malathion	1.4 (1.1, 1.7)	1.2 (1.0, 1.5)	1.2 (1.0, 1.4)	0.56
Parathion	1.5 (1.2, 1.9)	1.2 (0.9, 1.5)	1.2 (1.0, 1.5)	0.30
Phorate	1.3 (1.0, 1.6)	1.0 (0.9, 1.3)	1.0 (0.9, 1.2)	0.22
Terbufos	1.4 (1.1, 1.7)	1.2 (1.0, 1.4)	1.0 (0.8, 1.2)	0.02
Trichlorfon	1.7 (0.7, 4.1)	[f]	[f]	[f]
Pyrethroid insecticides	1.2 (1.0, 1.5)	1.2 (1.0, 1.4)	0.9 (0.8, 1.1)	0.09
Permethrin (for animals)	1.2 (1.0, 1.6)	1.1 (0.9, 1.4)	1.0 (0.8, 1.3)	0.48
Permethrin (for crops)	1.2 (0.9, 1.6)	1.3 (1.0, 1.7)	0.9 (0.7, 1.1)	0.04

Abbreviations: 2,4-D, (2,4-dichlorophenoxy)acetic acid; 2,4,5-T, (2,4,5-trichlorophenoxy)acetic acid; 2,4,5-TP, (*RS*)-2-(2,4,5-trichlorophenoxy)propionic acid; CI, confidence interval; DDT, 1,1,1-trichloro-2,2-bis(4-chlorophenyl)ethane; EPTC, *S*-ethyl dipropyl(thiocarbamate); OR, odds ratio; POST, post-enrollment; PRE-B, pre-enrollment both; PRE-E, pre-enrollment enrollment only.

[a]See footnote a at the bottom of Table S1 for a description of the three case groups. [b]Male private pesticide applicators who did not use each pesticide class or specific pesticide were the reference. [c]Adjusted for age at enrollment (modeled with a cubic polynomial), ever diagnosed with diabetes, education level, and state of residence. [d]Differences among case-group-specific ORs tested via Wald χ^2 tests. [e]Benomyl is also included in carbamates. [f]OR (95% CI) and *p* for difference not shown because fewer than five PRE-B or POST cases ever personally mixed or applied trichlorfon.

References

Cole SR, Hernán MA. 2008. Constructing inverse probability weights for marginal structural models. Am J Epidemiol 168:656-664.

Hernán MA, Brumback B, Robins JM. 2000. Marginal structural models to estimate the causal effect of zidovudine on the survival of HIV-positive men. Epidemiology 11:561-570.

Hernán MA, Hernández-Díaz S, Robins JM. 2004. A structural approach to selection bias. Epidemiology 15:615-625.

Hernán MA, Robins JM. 2006. Estimating causal effects from epidemiological data. J Epidemiol Community Health 60:578-586.

Muller KE, Fetterman BA. 2002. Regression and ANOVA: An Integrated Approach Using SAS Software. Cary, NC:SAS Institute Inc.

Robins JM. 1998. Marginal structural models. In: 1997 Proceedings of the American Statistical Association, Section on Bayesian Statistical Science. Alexandria, VA:American Statistical Association, 1–10.

Robins JM, Hernán MA, Brumback B. 2000. Marginal structural models and causal inference in epidemiology. Epidemiology 11:550-560.

Sato T, Matsuyama Y. 2003. Marginal structural models as a tool for standardization. Epidemiology 14:680–686.

Stokes, ME, Davis CS, Koch GG. 2000. Categorical Data Analysis Using the SAS System. 2nd ed. Cary, NC:SAS Institute Inc.
